# Protective mechanisms of a microbial oil against hypercholesterolemia: evidence from a zebrafish model

**DOI:** 10.3389/fnut.2023.1161119

**Published:** 2023-06-26

**Authors:** Adnan H. Gora, Saima Rehman, Jorge Dias, Jorge M. O. Fernandes, Pål A. Olsvik, Mette Sørensen, Viswanath Kiron

**Affiliations:** ^1^Faculty of Biosciences and Aquaculture, Nord University, Bodø, Norway; ^2^SPAROS Lda, Olhão, Portugal

**Keywords:** DHA, EPA, cardiovascular disease, plasma lipidomics, RNA seq, bioactive compounds

## Abstract

A Western diet elevates the circulating lipoprotein and triglyceride levels which are the major risk factors in cardiovascular disease (CVD) development. Consumption of long-chain omega-3 fatty acids can stall the disease progression. Although these fatty acids can significantly impact the intestine under a hypercholesterolemic condition, the associated changes have not been studied in detail. Therefore, we investigated the alterations in the intestinal transcriptome along with the deviations in the plasma lipids and liver histomorphology of zebrafish offered DHA- and EPA-rich oil. Fish were allocated to 4 dietary treatments: a control group, a high cholesterol group and microbial oil groups with low (3.3%) and high (6.6%) inclusion levels. We quantified the total cholesterol, lipoprotein and triglyceride levels in the plasma. In addition, we assessed the liver histology, intestinal transcriptome and plasma lipidomic profiles of the study groups. The results suggested that higher levels of dietary microbial oil could control the CVD risk factor indices in zebrafish plasma. Furthermore, microbial oil-fed fish had fewer liver vacuoles and higher mRNA levels of genes involved in β-oxidation and HDL maturation. Analyses of the intestine transcriptome revealed that microbial oil supplementation could influence the expression of genes altered by a hypercholesterolemic diet. The plasma lipidomic profiles revealed that the higher level of microbial oil tested could elevate the long-chain poly-unsaturated fatty acid content of triglyceride species and lower the concentration of several lysophosphatidylcholine and diacylglycerol molecules. Our study provides insights into the effectiveness of microbial oil against dyslipidemia in zebrafish.

## 1. Introduction

Disorders of the heart and blood vessels are grouped under the term cardiovascular diseases (CVDs). According to the World Health Organization, in 2019, about 18 million global deaths were due to CVDs, and most of such mortalities were witnessed in Asia and Europe (1, 2). The primary risk factor that instigates the development of CVDs is unhealthy food, the consumption of which can cause an imbalance in blood lipoprotein species (3). Lipoproteins are the primary carriers of cholesterol and altered lipoprotein levels are consequences of, among other factors, excess cholesterol consumption (4). Circulating cholesterol and lipoprotein levels can be restored with medication that is effective in hampering the intestinal absorption of excess dietary lipids (5, 6). However, the use of drugs, though effective, may induce severe side effects (7, 8). For instance, orlistat, a well-known pancreatic and gastric lipase inhibitor that prevents the absorption of lipids can, in some patients, induce steatorrhea and vitamin deficiency (9). On the other hand, statins which are used to inhibit cholesterol biosynthesis can cause muscle pain (10). Ezetimibe obstructs the absorption of cholesterol by interfering with the Niemann-Pick C1-Like 1 transporter for cholesterol absorption in the intestine. However, clinical observation of hepatitis has been associated with ezetimibe consumption (7). The side effects of the currently employed drugs indicate the need to identify alternate approaches to stall the progression of CVDs. Dietary bioactive compounds can be used to manage hypercholesterolemia because they act directly on the intestine (11). Furthermore, certain fatty acids can lower the risk of CVDs by increasing the proportion of small high density lipoprotein (HDL) particles, reducing the overload of cholesterol in them and elevating the cholesterol efflux capacity (efficiently performed by small HDL) of the particles (12, 13). Among these fatty acids, the long-chain omega-3 fatty acids, especially eicosapentaenoic acid (EPA, 20:5 *n* − 3) and docosahexaenoic acid (DHA, 22:6 *n* − 3), are believed to be effective in controlling CVDs (14).

Eicosapentaenoic acid and DHA can exert anti-atherogenic effects by lowering the circulating triacylglycerol (TAG) content (15), which is an independent risk factor that triggers the development of CVDs (16). EPA and DHA-rich fish oil is known to reduce the total cholesterol (17) and low-density lipoprotein (LDL) cholesterol content and increase the circulating high-density lipoprotein (HDL) cholesterol content (18) in the blood without affecting the size of HDL particles (19). However, HDL is a heterogenous population of particles and comprise two major subclasses, namely HDL2 which are larger and less dense compared to HDL3 particles which are smaller in size (20). The quantity of the total HDL cholesterol is dominated by HDL2 compared to HDL3 which is more efficient at reverse cholesterol transport (21). It must be noted that n-3 PUFA supplementation induces an increase in the cholesterol content of the HDL2 and a reduction in the cholesterol of HDL3 (19, 22). Interestingly, there exists an inverse relationship between high-density lipoprotein cholesterol (HDL-C) and the incidence of CVDs (23). HDL particles have the ability to acquire cholesterol from peripheral tissues and transfer it to the liver where it is converted to bile, a process known as reverse cholesterol transport (RCT) (24). One of the key players in RCT activity is hepatic scavenger receptor class B member 1 (SR-B1), but the conformation of apolipoprotein A-I (APOA1) influences HDL binding to SR-B1 (25). Another player in RCT activity is lecithin-cholesterol acyltransferase (LCAT). LCAT catalyzes the conversion of free cholesterol to esterified cholesterol, leading to the maturation of HDL particle. Mature HDL incorporates cholesterol from cells and tissues through its interaction with the SR-BI (to a lesser extent), ATP-binding cassette sub-family G member 1 (ABCG1) and ABCA1 and passive diffusion and transfers cholesterol to the liver *via* its interaction with SR-B1 (26–28). Dietary DHA and EPA impact the circulating HDL and SR-BI and LCAT activities, as observed in different animal models (29, 30). Dietary n-3 PUFAs are known to have a positive correlation with liver SR-B1 expression and the associated RCT (12) and lipids rich in EPA and DHA can interact better with SR-B1 (31).

Furthermore, these omega-3 fatty acids can alter CVD predictors, like Castelli I, Castelli II, atherogenic coefficient and atherogenic index (28, 32) which are considered better markers of CVD progression than absolute lipoprotein or lipid values (33–35). Another important mechanism by which these LC-PUFAs can exert their beneficial effect is by altering the circulating lipid species, which can be identified by studying plasma lipidome. Lipidomic studies have indicated that LC-PUFA consumption can selectively reduce the content of C12, C14 and C16 fatty acids (36) and increase LC-PUFA content of TAGs (37, 38).

The impact of omega-3 fatty acids on lipid metabolism has been revealed by studies that focused on alteration in plasma and liver lipids. Understanding the effect of DHA- and EPA-rich diet on the intestine is essential because it is the primary site where lipid absorption and packaging into chylomicrons and other lipoproteins take place (39). Nevertheless, the ability of EPA and DHA to manage dyslipidemia *via* their influence on the intestine has seldom been studied using appropriate models. To address this need, we investigated the effects of a microbial oil rich in EPA and DHA on the intestine transcriptome. *Schizochytrium* has been investigated in previous studies for its ability to reduce total cholesterol and LDL cholesterol levels (40, 41). Like fish oil (EPA:14.9%, DHA 13% of total fatty acids), *Schizochytrium* oil is also rich in n-3 PUFAs (EPA 15%, DHA 39% of total fatty acids). However, in contrast to fish oil it has a higher content of saturated fatty acids (42, 43). Zebrafish model was used as the experimental species (44) in this study because we wanted to model diet-induced dyslipidemia. Zebrafish is an emerging model species for studying diet-induced dyslipidemia. In terms of morphology as well as the features that aid in digestion and absorption, zebrafish intestine resembles that of mammals (45, 46). A recent study has identified the orthologues of mammalian lipoproteins, and dietary lipids can modulate their expression (47). Therefore, we employed a diet-induced hypercholesterolemia model of zebrafish that simulates the dysregulated lipid metabolism in vertebrates to explore the effects of microbial oil rich in EPA and DHA on the intestine transcriptome. We also studied the plasma lipidomic profiles to understand the changes in the plasma of zebrafish that were fed EPA- and DHA-rich microbial oils. Additionally, we studied the plasma lipidomic profiles to understand the changes in the plasma of zebrafish that were fed EPA- and DHA-rich microbial oils.

## 2. Materials and methods

### 2.1. Experimental fish

The approval for the conduct of this study was obtained from the Norwegian Animal Research Authority (FDU ID: 22992). All the experimental procedures involving animals were in accordance with the EU Directive 2010/63 on the use of animals for scientific purposes. To obtain the 240 male zebrafish (6-month-old) required for the study, adult zebrafish (AB line) were bred in-house at the zebrafish facility of Nord University, Norway, following standard protocols (48). Zebrafish eggs were obtained by breeding sexually mature males and females. Fish in five tanks were used for breeding, and in each of these tanks there were 15 males and 30 females. They were community bred and 300–400 eggs were obtained from each tank. The eggs were maintained in E3 medium and incubated at 28°C in an incubator until hatching, i.e., around 50 h post-fertilization. From 4 to 14 days post-fertilization, the larvae were fed (*ad libitum*) the commercial micro diet Zebrafeed^®^ of SPAROS Lda, Olhão, Portugal (< 100 μm particle size) and *Artemia nauplii*. From 15 days post-fertilization (advanced larval stage) onwards, the fish were fed micro diets of 100–200 μm particle size (Zebrafeed^®^). When the fish were 5-month-old, they were randomly distributed into 24 tanks (6 tanks per treatment group) on a freshwater flow-through system (Zebtec Toxicological Rack, Tecniplast, Varese, Italy) with 3.5 l tank capacity. We selected only male zebrafish for our study as previous reports have indicated sex-based differences in lipid metabolism (49, 50). The stocking density was ten fish per tank. The experimental fish were acclimatized in the flow-through system for 4 weeks during which period they were fed the experimental control diet. The water temperature in the tanks was 28 ± 0.5°C, and the water flow rate was 2.5 l/h. The dissolved oxygen in the tanks ranged between 7 and 8 ppm (oxygen saturation > 85%). A 14 l:10D photoperiod was maintained throughout the experiment.

### 2.2. Diets and feeding regimen

The four experimental diets were prepared by SPAROS Lda. (Supplementary Table 1): one control diet and three high-cholesterol diets. A standard zebrafish diet without the purified cholesterol served as the control diet, CT. The high cholesterol (HC) diet had 5.1% (*w*/*w*) inclusion of purified cholesterol and 6.6% inclusion of soybean oil. We selected the cholesterol level based on previous studies with zebrafish (51, 52). Two diets were prepared by incorporating the microbial oil derived from *Schizochytrium* sp. (Veramaris, Delft, Netherlands): HCA1 and HCA2 in which the inclusion level of the microbial oil was 3.1 and 6.6%, respectively; replacing 3.5 and 6.6% of soybean oil. The EPA:DHA ratio in the CT and HC diets was 1.45, whereas the ratio in the HCA1 and HCA2 diets were 0.49 and 0.45, respectively (Supplementary Table 1). The daily feeding rate was 4% of the total biomass in the tanks. This ration was split into three feeding events per day—at 08:00, 13:00, and 18:00—to allow the fish to consume the feed instantly so that no leftover feed remained in the tanks during the experimental period of 12 weeks.

### 2.3. Sampling

At the end of the feeding trial, fish were euthanized by immersion (for 3 min) in 200 mg L^−1^ of tricaine methanesulfonate (Cat. Number: E10521, SigmaAldrich, Saint Louis, U.S.), which was buffered with 200 mg L^−1^ of sodium bicarbonate (Cat. Number: S5761, SigmaAldrich). The blood collected by tail ablation (53) was centrifuged at 5000 *g* for 10 min at 4°C to obtain the plasma. There are seven distinct regions in the gut of zebrafish based on their gene expression profiles. The mid-region of the gut has high expression of genes like *fatty acid binding protein 2, intestinal* (*fabp2*), *apolipoprotein A-Ia* (*apoa1a*) and apolipoprotein A-IV a (*apoa4a*) that are involved in lipid metabolism (54). It has also been reported that the main fatty acid transporter gene *thrombospondin receptor* (*cd36*) and cholesterol homeostasis genes like *apolipoprotein Bb, tandem duplicate 1* (*apobb.1*)*, cytochrome P450, family 7, subfamily A, polypeptide* 1 (*cyp7a1*)*, apolipoprotein Ba* (*apoba*)*, cholesteryl ester transfer protein* (*cetp*) and *apolipoprotein a-Ib* (*apoa1b*) have significantly higher expression in the mid-intestine region of zebrafish compared to the anterior and posterior regions (55). Therefore, mid-intestine was considered for RNA Seq. The whole liver was also dissected and snap-frozen in liquid nitrogen and stored at − 80°C for qPCR study.

### 2.4. Plasma cholesterol and triglyceride estimation

The total, LDL, and HDL cholesterol levels in the plasma were estimated using the HDL and LDL/VLDL Cholesterol Assay Kit (Cat. Number: ab65390, Abcam, Cambridge, United Kingdom). Total triacylglycerides in the plasma were estimated using the Triglyceride Assay Kit (Cat. Number: ab65336, Abcam), according to the manufacturer’s instructions. Each treatment consisted of six replicates and each replicate was a pool of plasma from 6 fish per tank. The CVD risk indices were calculated using the following four equations (56):


(1) CastelliIindex=Total cholesterol/HDLcholesterol.

(2) CastelliIIindex=LDLcholesterol/HDLcholesterol.

(3) Atherogenic index=log(TAG/HDL).

(4) $$\mathrm{Atherogenic coefficient}=\left(\begin{array}{l} \mathrm{Total cholesterol}\\ -\mathrm{HDL}\;\mathrm{cholesterol} \end{array}\right)/\mathrm{HDL}\;\mathrm{cholesterol}.$$

### 2.5. Intestine RNA-sequencing and bioinformatic analyses

To extract total RNA, the frozen intestine samples were briefly homogenized in QIAzol lysis reagent (Cat. Number: 79306, Qiagen, Hilden, Germany) at 6500 rpm for 2 × 20 s in a Precellys 24 homogenizer (Cat. Number: P000669-PR240-A, Bertin Instruments, Montigny-le-Bretonneux, France). RNA was extracted from the tissue homogenate using Direct-zol^™^ RNA MiniPrep kit (Cat. Number: R2052, Zymoresearch, CA, United States) following the manufacturer’s instructions. The RNA concentration and integrity were checked using Qubit™ RNA Broad Range (BR) Assay Kit (Cat. Number: Q10210, Thermo Fisher Scientific, Waltham MA, United States) with a Qubit 4 Fluorometer (Cat. Number: Q33238) and Tape Station 2,200 (Cat. Number: G2964AA, Agilent Technologies, Santa Clara, CA, United States). Only the RNA samples that had RIN value > 7 were used to construct RNA-Seq libraries. Library preparation and sequencing was performed by Novogene Europe (Cambridge, United Kingdom). The mRNA was purified from total RNA using poly-T oligo-attached magnetic beads. After fragmentation, the first strand cDNA was synthesized using random hexamers followed by the second strand cDNA synthesis. The libraries were end repaired, A-tailed, adapter ligated, size selected, amplified, and finally purified. The libraries were quantified by Qubit and real-time PCR. Furthermore, a Bioanalyzer (Agilent technologies) detected the size distribution. The barcoded libraries were then pooled and loaded on the Illumina NovaSeq 6,000 Sequencing system (Illumina, San Diego, CA, United States) to obtain 150 bp paired end reads. For each sample, a minimum of 20 million paired raw reads were obtained, with an average of 22.3 million reads per sample (Supplementary Table 2). The quality of the raw reads was assessed using the *fastQC* command. Low quality reads (Phred quality score, Q < 30) were filtered from the raw reads using the *fastp* software (57). The filtered reads were then aligned to the reference zebrafish genome downloaded from NCBI (release 106) after indexing using HISAT2, version 2.2.1 (58). The average mapping percentage for the whole dataset was 87.5%. The reads were annotated using *featureCounts* to obtain the read counts of each gene (59). Differential expression analyses of the genes in the treatment groups were performed using the R package *DESeq2* (version 1.30.0). Transcripts with an absolute Log_2_ fold change of ≥ 1 and an adjusted *p* value of < 0.05 (Benjamini-Hochberg multiple test correction method) were considered significantly differentially expressed and were used for gene ontology (GO) and KEGG pathway analyses. The GO enrichment was performed with Database for Annotation, Visualization and Integrated Discovery (*DAVID*) version 6.8 (60) and *clusterProfiler* package (version 3.18.0) in R. The R packages *ggplot2* (version 3.3.3), *pheatmap* (version 1.0.12) and *GOplot* (version 1.0.2) were employed to visualize the data. GO term-gene networks were generated using *Cytoscape* 3.8.2 (61).

### 2.6. Liver histomorphometry

The liver from the experimental fish (*n* = 9–12 per group) was dissected and immediately fixed in 3.7% (*v*/*v*) phosphate-buffered formaldehyde solution (pH 7.2) at 4°C for 24 h. Standard histological procedures were employed for dehydration, processing, and paraffin embedding, as described by Bancroft and Gamble (62). The paraffin blocks thus prepared were sectioned using a microtome (Microm HM355S, MICROM International GmbH, Walldorf, Germany). Four micrometer thick sections were cut and mounted on SuperFrost^®^ slides (Menzel, Braunschweig, Germany). A robot slide stainer Microm HMS 760 × (MICROM International GmbH) was used to stain the liver sections with hematoxylin (Cat. Number: H9627, SigmaAldrich) and eosin (Cat. Number: 861006, SigmaAldrich). Light microscopy photomicrographs were taken with Leica DM3000 LED microscope (Leica Camera AG, Wetzlar, Germany) fitted with Leica MC 190HD camera (Leica Camera AG). The software *ImageJ* (63) was used to analyze the images. Liver vacuolation was assessed by evaluating two parameters—average vacuole area and average vacuole number in randomly selected areas of the liver (64). Shapiro–Wilk and Bartlett’s tests were employed to confirm normality and homoscedasticity of the data, respectively. One-way ANOVA was employed where the assumptions were met. In the case of non-parametric data, statistical differences were evaluated using the Kruskal–Wallis test.

### 2.7. Hepatic gene expression analysis

We performed qPCR to understand the effect of the supplementation of microbial oil on hepatic gene expression, focusing on genes linked to (i) fatty acid β-oxidation in mitochondria and peroxisomes (*cpt1aa*, *acaa*), (ii) intracellular lipid droplets (*plin2*), and (iii) HDL metabolism (*lcat*, *scarb1* and *abca1a*). One μg of total RNA from each sample was reverse transcribed using the QuantiTect reverse transcription kit (Cat. Number: 205311, Qiagen), according to the manufacturer’s instructions. The cDNA was further diluted ten times with nuclease-free water and used as a qPCR template. The qPCR reactions were carried out using SYBR green (Cat. Number: 04707516001, Roche Holding AG, Basel, Switzerland) in a LightCycler^®^ 96 Real-Time PCR System (Cat. Number: 05815916001, Roche Holding). Relative expression of selected genes was determined based on the geometric mean of previously reported reference genes (*actb1*, *eef1a1l1* and *rpl13α*) (65) following the protocol described by Livak and Schmittgen (66). We designed the primers for the selected genes using the Primer-BLAST tool in NCBI. The primers were then checked for secondary structures such as hairpin, repeats, self and cross dimers by *NetPrimer* (Premier Biosoft, Palo Alto, United States). The primers for the target genes are listed in Supplementary Table 3. The efficiency of all primers was confirmed by the method described by Pfaffl (67). The data were checked for normality (Shapiro–Wilk test) and homoscedasticity (Bartlett’s test), based on which, the statistical difference was determined by one-way ANOVA or Kruskal-Wallis test.

### 2.8. Plasma lipidome profiling

Lipidome profiling was carried out by MS-Omics (Vedbæk, Denmark). Plasma samples were mixed 1:9 with isopropanol containing 0.1 M benzothiazolone hydrazone and internal standards before transferring to SpinX filters. The samples were then mixed in a vortex for 60 s and left at room temperature for 10 min before placing in a − 20°C freezer overnight. The following day, samples were left at room temperature for 30 min before centrifuging (14,000 rpm/5°C/2 min). Finally, the samples were mixed with eluent. The analysis was carried out using a Thermo Scientific Vanquish LC (Thermo Fisher Scientific) coupled to Thermo Q Exactive HF MS (Thermo Fisher Scientific). The lipids extracted from the samples were ionized in positive and negative ionization mode using an electrospray ionization interface. Then, chromatographic separation of lipids was carried out on a Waters^®^ ACQUITY Charged Surface Hybrid (CSH^™^) C18 column (2.1 × 100 mm, 1.7 μm; Waters Corporation, Milford, United States) at 55°C. The mobile phases consisted of (A) acetonitrile/water (60:40) and (B) isopropanol/acetonitrile (90:10), both with 10 mM ammonium formate and 0.1% (*v*/*v*) formic acid. Lipids were eluted in a two-step gradient by increasing B in A from 40 to 99% over 18 min, and the flow rate was 0.4 ml/min. The obtained peak areas were extracted using Compound Discoverer 3.2 (Thermo Fisher Scientific). Thereafter, compounds were identified at four levels; Level 1: identification by retention times (compared against in-house authentic standards), accurate mass (with an accepted deviation of 3 ppm), and MS/MS spectra; Level 2a: identification by retention times (compared against in-house authentic standards), accurate mass (with an accepted deviation of 3 ppm); Level 2b: identification by accurate mass (with an accepted deviation of 3 ppm), and MS/MS spectra; Level 3: identification by accurate mass alone (with an accepted deviation of 3 ppm). The obtained lipidome data were analyzed employing *MetaboAnalyst 5.0* (68). The data were log transformed and auto-scaled (mean-centered and divided by the standard deviation of each variable; Supplementary Figure 1) before downstream analyses. Principal component analysis was performed using the *mixomics* package in *R 4.2.1* to understand the differential clustering of the study groups. A |Log_2_ fold change| ≥ 1 and a *p* value of < 0.05 were considered to identify the significantly altered lipid species. The over representation analysis (ORA) based on the significantly altered lipids was performed using a reference of 1,072 sub chemical class of lipid sets. The ORA, which employs the hypergeometric test, was performed using the differentially abundant lipid species. A *p* value cut-off of < 0.05 and a minimum lipid species count ≥ 2 for each lipid set were considered as significantly enriched lipid classes. *Cytoscape 3.9.0* and *ggplot2* package in *R 4.2.1* were employed to present the data.

## 3. Results

### 3.1. Microbial oil lowered the cholesterol and TAG levels in the plasma

**Figure 1:**
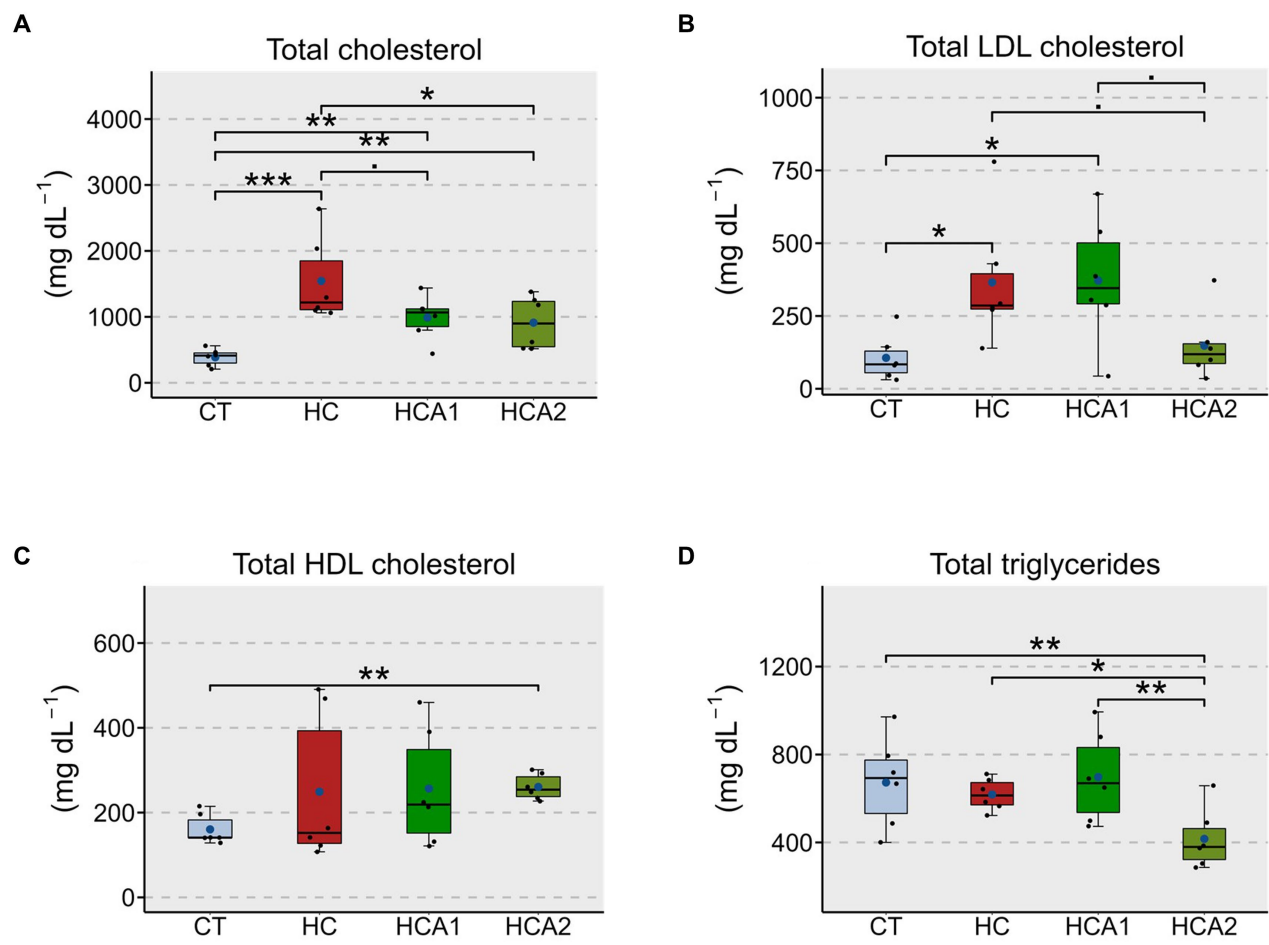
Alteration of the plasma lipids in zebrafish fed different experimental diets for a period of 12 weeks. Boxplots show total cholesterol **(A)**, LDL cholesterol **(B)**, HDL cholesterol **(C)** and total triglyceride **(D)** contents in the plasma of the fish. Blue dots inside boxplots indicate the mean values of the corresponding groups. CT-fish fed the control diet; HC-fish fed the high cholesterol diet (5.1% inclusion); HCA1-fish fed microbial oil (3.1% inclusion); HCA2-fish fed microbial oil (6.6% inclusion). ^***^p < 0.001, ^**^p < 0.01, ^*^p < 0.05, and ^•^*p* < 0.1. Each treatment group consisted of six biological replicates.

We examined the effect of two levels of dietary microbial oil (HCA1-low level and HCA2-high level) on the circulating cholesterol and TAG levels in zebrafish. Total cholesterol (values presented as mg dL^-1^) of the HC group (1544.6 ± 590.9; a model of hypercholesterolemia) was significantly higher (*p* < 0.001) compared to the CT group (386.9 ± 130.1; Figure 1A). The HCA2 group (912.0 ± 400.4) had significantly (*p* < 0.05) lower total cholesterol compared to the HC group. However, the total cholesterol levels of the HCA1 (988.9 ± 308.4) and HCA2 groups were significantly higher compared to the CT group. While the HC group had a significantly (*p* < 0.05) higher plasma LDL cholesterol (values presented as mg dL^-1^, 365.3 ± 203.5) compared to the CT group (106.0 ± 72.8), the LDL cholesterol level of HCA2 group (147.7 ± 107.9) was not significantly different (*p* > 0.05) from the CT group (Figure 1B). The HCA1 group also had significantly (*p* < 0.05) higher level of LDL cholesterol (371.6 ± 198.27) compared to CT. A significantly higher (*p* < 0.01) amount of HDL cholesterol (values presented as mg dL^-1^) was found in HCA2 group (260.5 ± 27.7) compared to the CT group (160.4 ± 32.7; Figure 1C). We also found that the HCA2 group had a higher proportion of esterified cholesterol content (0.85 ± 0.1) compared to the other groups though the increase was not statistically significant (Supplementary Figure 2). We further investigated the hypolipidemic effect of HCA2 diet by estimating the TAG levels (values presented as mg dL^-1^) in the different diet groups. The HCA2 group (416.2 ± 126.5) had a significantly (*p* < 0.05) lower level of triglycerides compared to the CT (675.0 ± 187.0), HC (618 ± 66.1) and HCA1 (697.0 ± 188.5) groups (Figure 1D).

**Figure 2:**
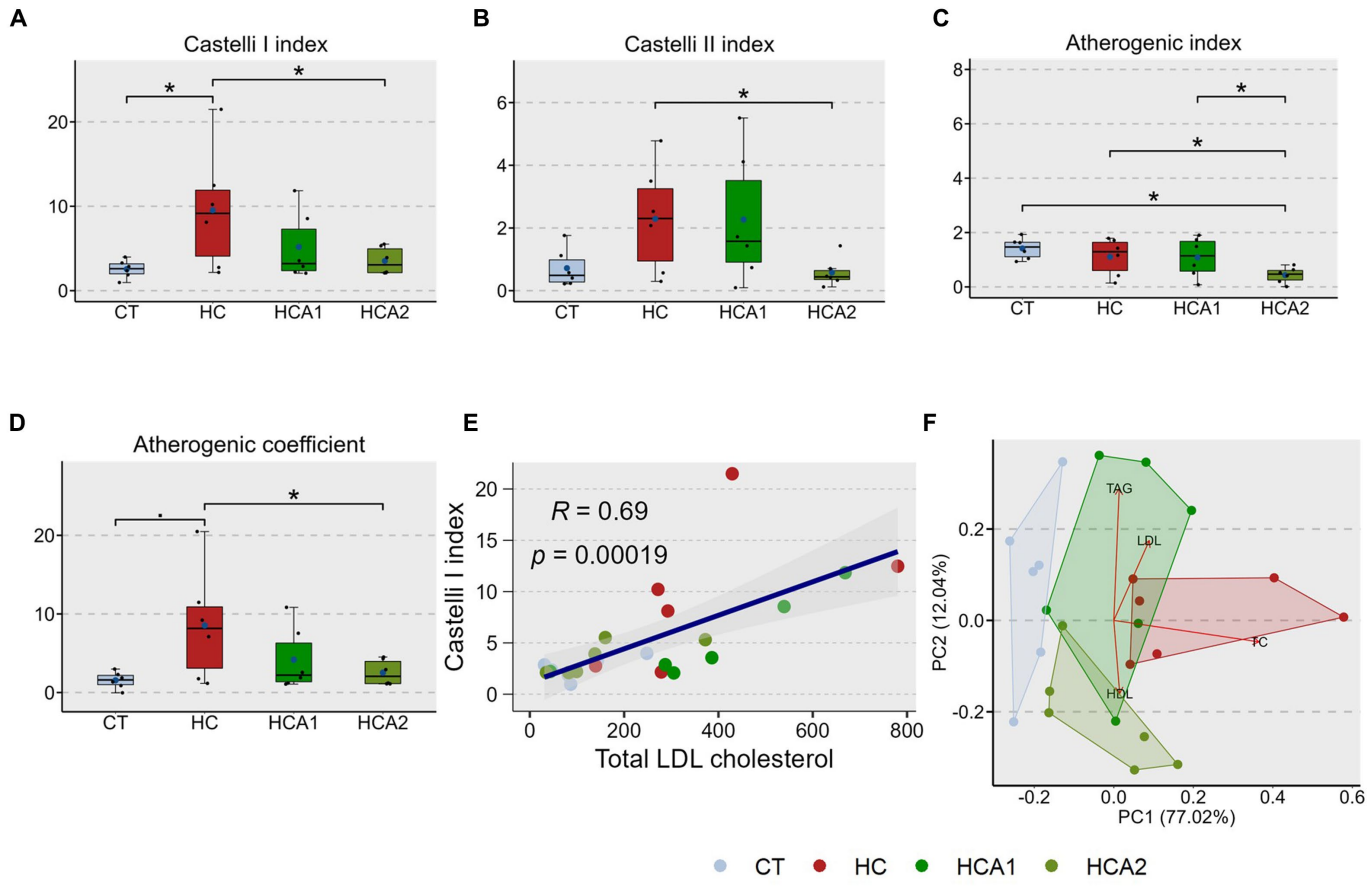
Alteration of the cardiovascular disease (CVD) risk indices in zebrafish fed different experimental diets for a period of 12 weeks. Boxplots show Castelli I index **(A)**, Castelli II index **(B)**, Atherogenic index **(C)** and Atherogenic coefficient **(D)** of plasma from the fish. Blue dots inside boxplots indicate the mean values of the corresponding groups. Scatter plot **(E)** shows the correlation between Castelli I index and total LDL cholesterol levels. Biplot **(F)** indicates the separation of the CT and HCA2 groups from the other 2 study groups and variables associated with each group. CT-fish fed the control diet; HC-fish fed the high cholesterol diet (5.1% inclusion); HCA1-fish fed microbial oil (3.1% inclusion); HCA2-fish fed microbial oil (6.6% inclusion). ^*^p < 0.05 and ^•^*p* < 0.1. Each treatment group consisted of six biological replicates.

Using the plasma TC, LDL cholesterol, HDL cholesterol and TAG values of the treatment groups, we estimated the different CVD risk indices: Castelli I index (Equation 1), Castelli II index (Equation 2), Atherogenic index, AI (Equation 3) and Atherogenic coefficient, AC (Equation 4). The HC group had a significantly higher Castelli I index (9.5 ± 7.1) compared to the CT group (2.5 ± 1.0; Figure 2A). The HCA2 group had significantly lower values for all four predictors (Castelli I = 3.52 ± 1.6, Castelli II = 0.57 ± 0.5, AI = 0.43 ± 0.3, AC = 2.52 ± 1.6) of cardiovascular disease (PCVD), compared to the HC group (Castelli II = 2.2 ± 1.7, AI = 1.10 ± 0.7, AC = 8.53 ± 7.1; Figures 2A–D). Furthermore, the HCA2 group had significantly lower atherogenic index compared to CT (1.41 ± 0.4) and HCA1 (1.08 ± 0.7) groups (Figure 2C). Correlation between plasma lipid species and PCVD is presented in Supplementary Figure 3A. HDL was found to have a negative correlation with PCVD. We also observed a significantly positive correlation (*R* = 0.69, *p* = 0.00019) between Castelli I index and LDL cholesterol content (Figure 2E). The correlation between TAG:HDL ratio and LDL cholesterol was also positive (*R* = 0.37) but not significant (*p* = 0.086; Supplementary Figure 3B). The principal component analysis biplot revealed a separation of the HC group from the CT and HCA2 groups along the principal component 1 (PC1) that captured 77% variability (Figure 2F). We also found a separation of the HC1 and HCA2 groups, predominantly driven by differences in the HDL and TAG levels of the two groups. The differences in the CT and HC groups are due to the total cholesterol content.

### 3.2. HCA2 diet altered the liver histomorphology and gene expression

**Figure 3:**
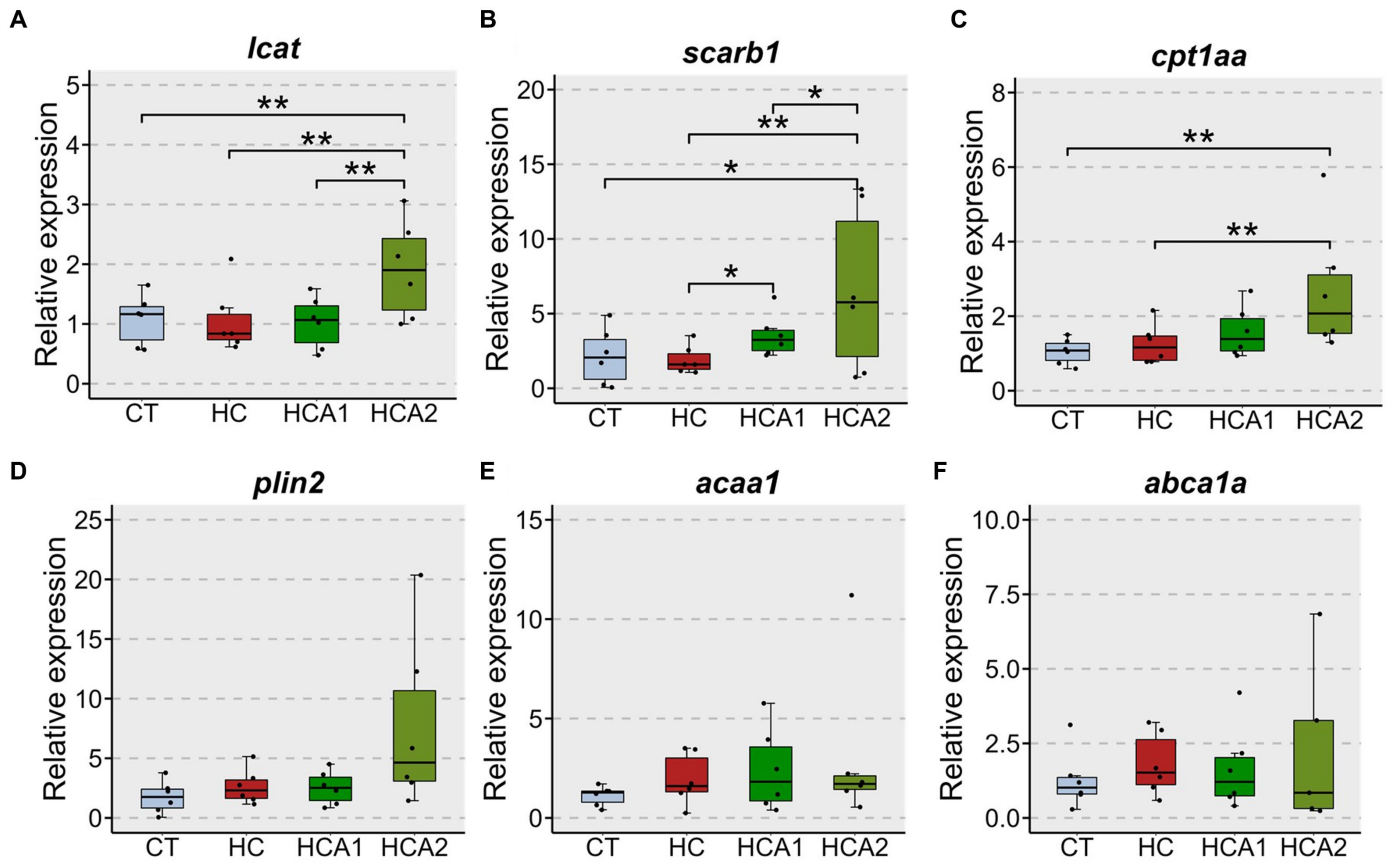
Relative expression of selected genes in the liver of zebrafish fed different experimental diets. *lecithin-cholesterol acyltransferase* (*lcat*) **(A)**; *scavenger receptor class B, member 1* (*scarb1*) **(B)**; *carnitine palmitoyltransferase 1Aa* (*cpt1aa*) **(C)**; *perilipin 2* (*plin2*) **(D)**; *acetyl-CoA acyltransferase 1* (*acaa1*) **(E)**; *ATP-binding cassette, sub-family A (ABC1)*, *member 1A* (*abca1a*) **(F)**. Black dots indicate the relative expression of the respective genes in each sample. CT-fish fed the control diet; HC-fish fed the high cholesterol diet (5.1% inclusion); HCA1-fish fed microbial oil (3.1% inclusion); HCA2-fish fed microbial oil (6.6% inclusion). ^**^p < 0.01 and ^*^p < 0.05. Each treatment group consisted of six biological replicates.

**Figure 4:**
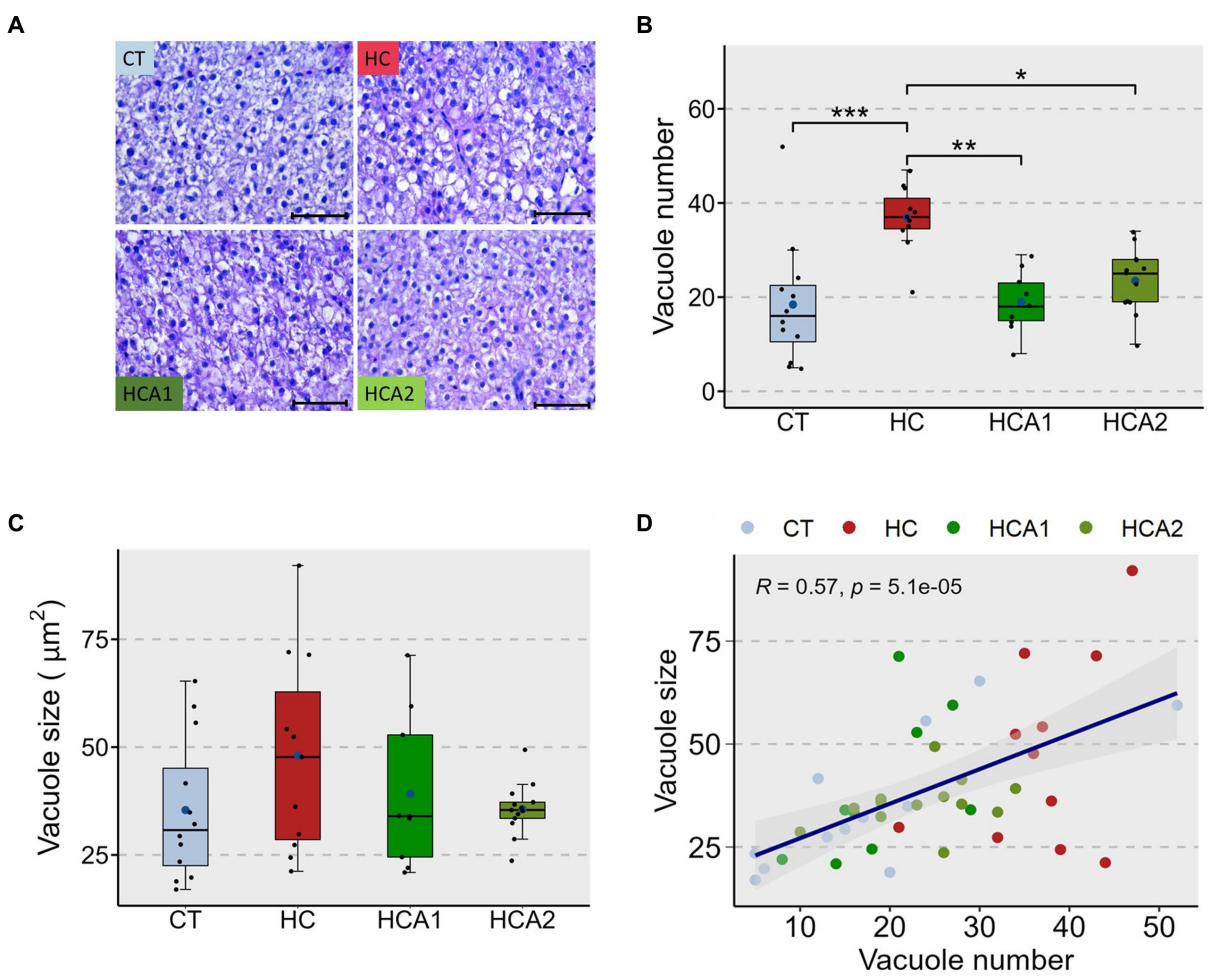
Histomorphology of the liver of zebrafish fed different experimental diets for a period of 12 weeks. Representative histological images **(A)** of the liver of zebrafish. Dot-plot shows average number **(B)** and average size **(C)** of vacuoles in the liver of fish fed control (CT) diet, high cholesterol (HC) diet, HC diet supplemented with lower (HCA1) or higher (HCA2) levels of microbial oil. The scatter plot **(D)** shows correlation between average vacuole number and average vacuole size in the liver of zebrafish. ^***^p < 0.001, ^**^p < 0.01 and ^*^p < 0.05. Each treatment group consisted of 9–12 biological replicates. Scale bar = 50 μm.

The relative expression of the *lcat* gene was increased by two-fold (*p* < 0.05) in the HCA2 group compared to the CT, HC and HCA1 groups (Figure 3A). The expression of *scarb1* in the HCA2 group increased 9-fold (*p* < 0.05) compared to the CT, HC and HCA1 groups (Figure 3B). We observed a two-fold increase (*p* < 0.05) in the mRNA level of the gene *cpt1aa* in the HCA2 group compared to the CT and HC groups (Figure 3C). However, we did not detect a statistically significant difference in the relative expression of *plin2*, *acaa* and *abca1a* (Figures 3D–F). We also investigated histological changes in the liver to understand the effect of different diets on vacuolization (Figure 4A). We observed a significantly higher number of vacuoles (Figure 4B) in the HC group compared to CT (*p* < 0.001). Furthermore, both HCA1 and HCA2 groups had significantly lower vacuole number (*p* < 0.01 and *p* < 0.05, respectively) compared to the HC group. Although, the mean vacuole size of the study groups was not significantly different (*p* > 0.05; Figure 4C), we detected a significant correlation (*R* = 0.57, *p* < 0.001) between vacuole size and number in the liver of the different diet groups (Figure 4D).

### 3.3. The intestinal transcriptome reflected diet-induced hypercholesterolemia in zebrafish

**Figure 5:**
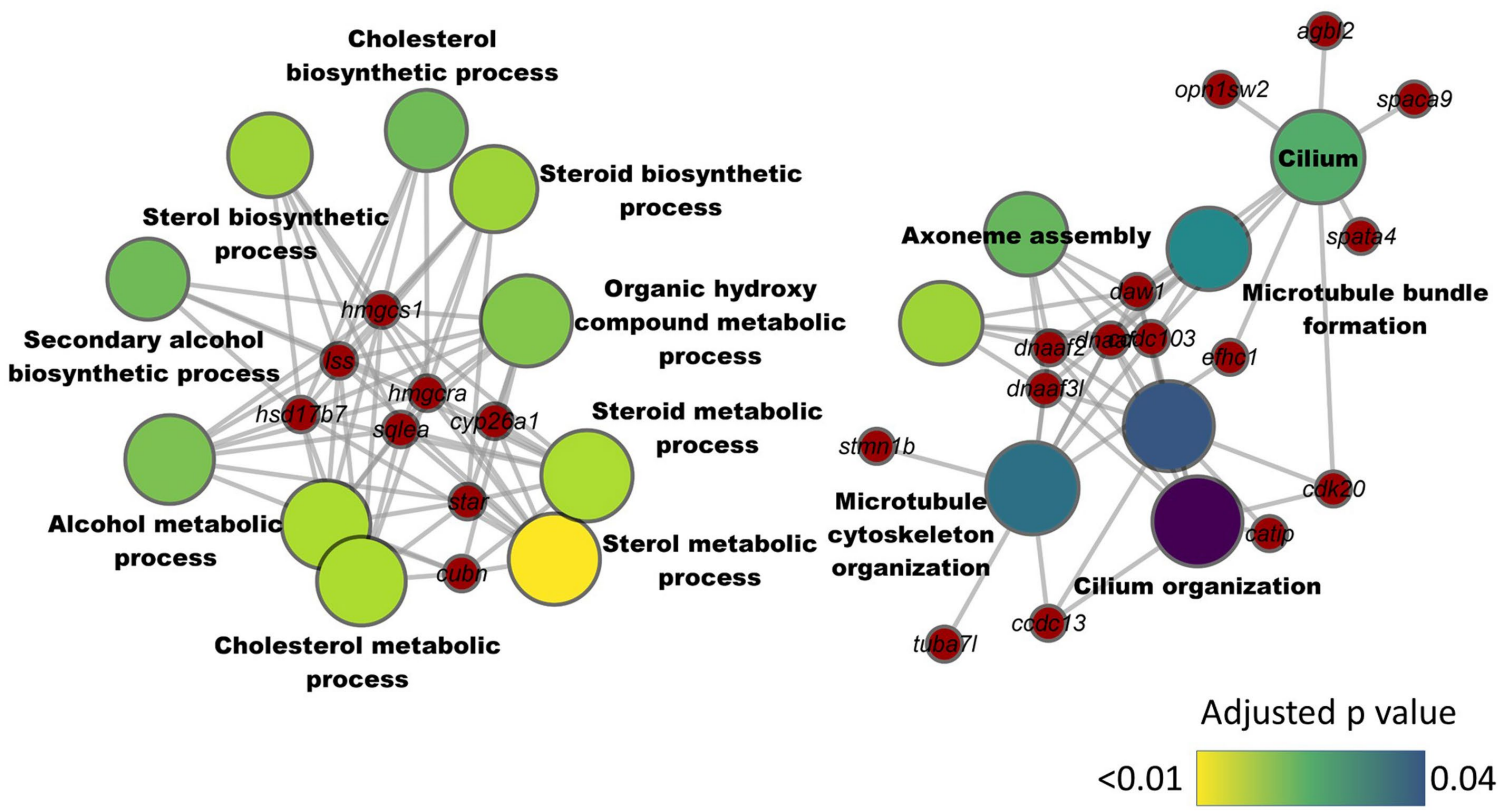
Network plot showing the link between enriched GO terms and the associated genes that were downregulated in the intestine of zebrafish fed high cholesterol diet. The significantly downregulated genes from the HC vs. CT comparison are written on the red nodes. Only the non-redundant GO terms are labelled in the network plot. For each GO term, the gradient color varies with the adjusted *p* value (Benjamini-Hochberg method). Adjusted *p* value (Benjamini-Hochberg method) < 0.05 and minimum gene count of 2 were set as cut-off parameters for each GO term.

We analyzed the intestinal transcriptome of zebrafish from the different treatment groups. The comparison between HC and CT groups revealed 164 differentially expressed genes (DEGs), of which 146 were downregulated and 18 were upregulated in the HC group (Supplementary Table 4). These included c*ytochrome P450, family 26, subfamily A, polypeptide 1* (*cyp26a1*), *cubilin* (*cubn*), *3-hydroxy-3-methylglutaryl-CoA reductase a* (*hmgcra*), *steroidogenic acute regulatory protein* and *3-hydroxy-3-methylglutaryl-CoA synthase 1* (*soluble; hmgcs1*). The KEGG pathway enrichment employing the downregulated DEGs revealed a significant suppression of the steroid biosynthesis pathway (Supplementary Table 5). Furthermore, the GO enrichment analysis based on the downregulated DEGs revealed significant enrichment of several GO terms in two separate clusters. One of the clusters was linked to cholesterol metabolism and included terms like cholesterol biosynthetic process, steroid metabolic process and steroid biosynthetic process (Figure 5). The second cluster included GO terms like microtubule cytoskeleton organization, cilium organization, microtubule bundle formation and axoneme assembly. The upregulated genes in the HC group were not found to significantly enrich any KEGG pathways or GO terms.

#### 3.3.1. Microbial oil altered the intestinal transcriptome of the zebrafish model of hypercholesterolemia

**Figure 6:**
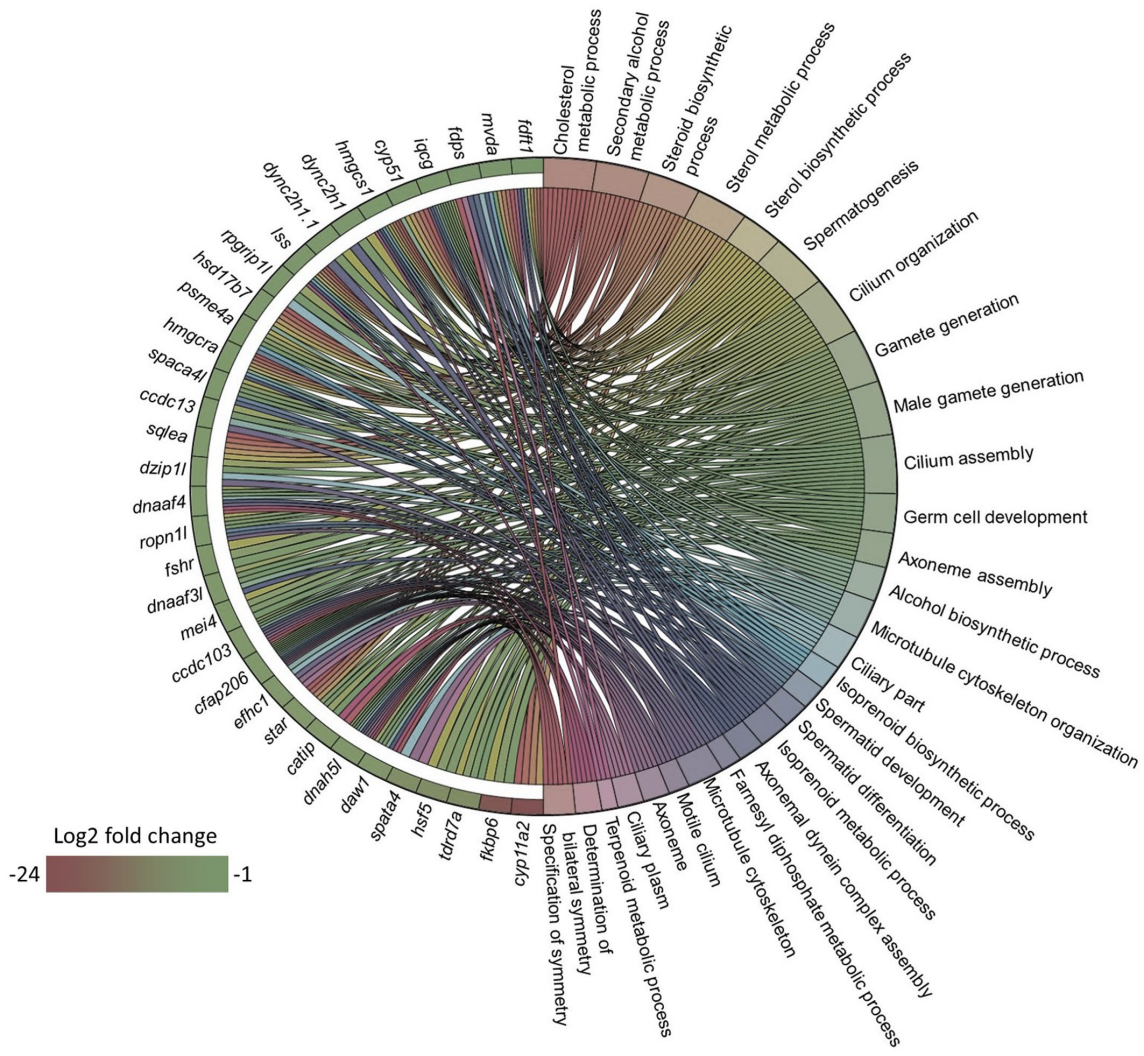
Gene ontology (GO) term enrichment based on the downregulated genes in the HCA1 group. Chord diagram showing the link between the enriched GO terms and the associated downregulated genes from the HCA1 vs. CT group comparison. Genes that were considered for the enrichment analyses are shown on the left half of the circle. The gradient color bar intensity varies with the Log_2_ fold change.

Since the microbial oil in the diet was able to lower the plasma total and LDL cholesterol levels in zebrafish, we wanted to understand if these effects can be associated with the intestinal transcriptome. We first compared the intestinal transcriptome of the HCA1 group with that of the CT group. This analysis revealed 177 DEGs, of which 15 were upregulated and 162 were downregulated in the HCA1 group (Supplementary Table 6). GO enrichment analysis employing the downregulated DEGs revealed terms like cholesterol biosynthetic process, steroid metabolic process, and steroid biosynthetic process, microtubule cytoskeleton and microtubule cytoskeleton organization (Figure 6).

**Figure 7:**
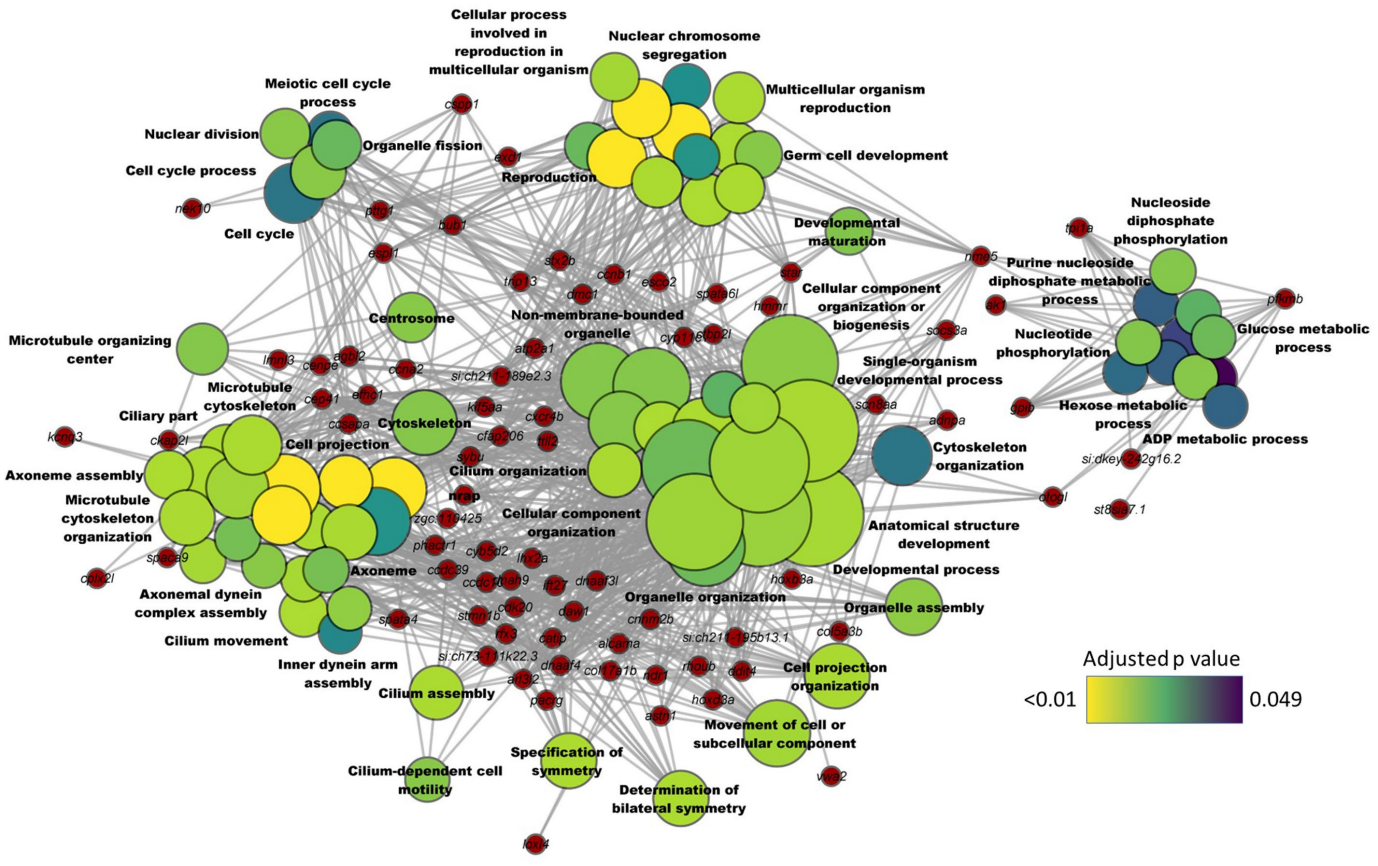
Network plot showing the link between enriched GO terms and the associated genes that were downregulated in zebrafish fed microbial oil (HCA2) supplemented diet. The significantly downregulated genes (HCA2 vs. CT) are written on the red nodes. Only the non-redundant GO terms are labelled and for each GO term, the gradient color varies with the adjusted p value (Benjamini-Hochberg method). Adjusted *p* value (Benjamini-Hochberg method) < 0.05 and minimum gene count of 2 were set as cut-off parameters for each GO term.

Downregulated DEGs-based KEGG pathway enrichment revealed a significant suppression of steroid biosynthesis and terpenoid biosynthesis (adjusted *p* value < 0.01) in the HCA1 group (Supplementary Table 7). These enriched GO terms and pathways were similar to the results of the comparison between the transcriptomes of the HC and CT groups. Given that the results of the HC vs. CT and HCA1 vs. CT comparisons were similar, we wanted to know if a higher level of the microbial oil can mitigate the effects of dietary high cholesterol. Hence, we performed the HCA2 vs. CT comparison. This analysis revealed 182 DEGs, of which 162 were downregulated and 20 were upregulated in the HCA2 group (Supplementary Table 8). The GO terms that were enriched by the downregulated DEGs included glucose metabolic process, ADP metabolic process, microtubule cytoskeleton organization, nuclear division and cell cycle process (Figure 7). However, we did not find any enriched GO terms linked to cholesterol metabolism. Steroid biosynthesis and terpenoid biosynthesis pathways were not enriched based on the downregulated DEGs in the HCA2 group. The differentially upregulated DEGs led to the enrichment of KEGG pathways like glutathione metabolism, drug metabolism-cytochrome p450 and metabolism of xenobiotic by cytochrome p450 (Supplementary Figure 4; Supplementary Table 9).

#### 3.3.2. HCA2 diet influenced the expression of genes involved in cholesterol biosynthesis

**Figure 8:**
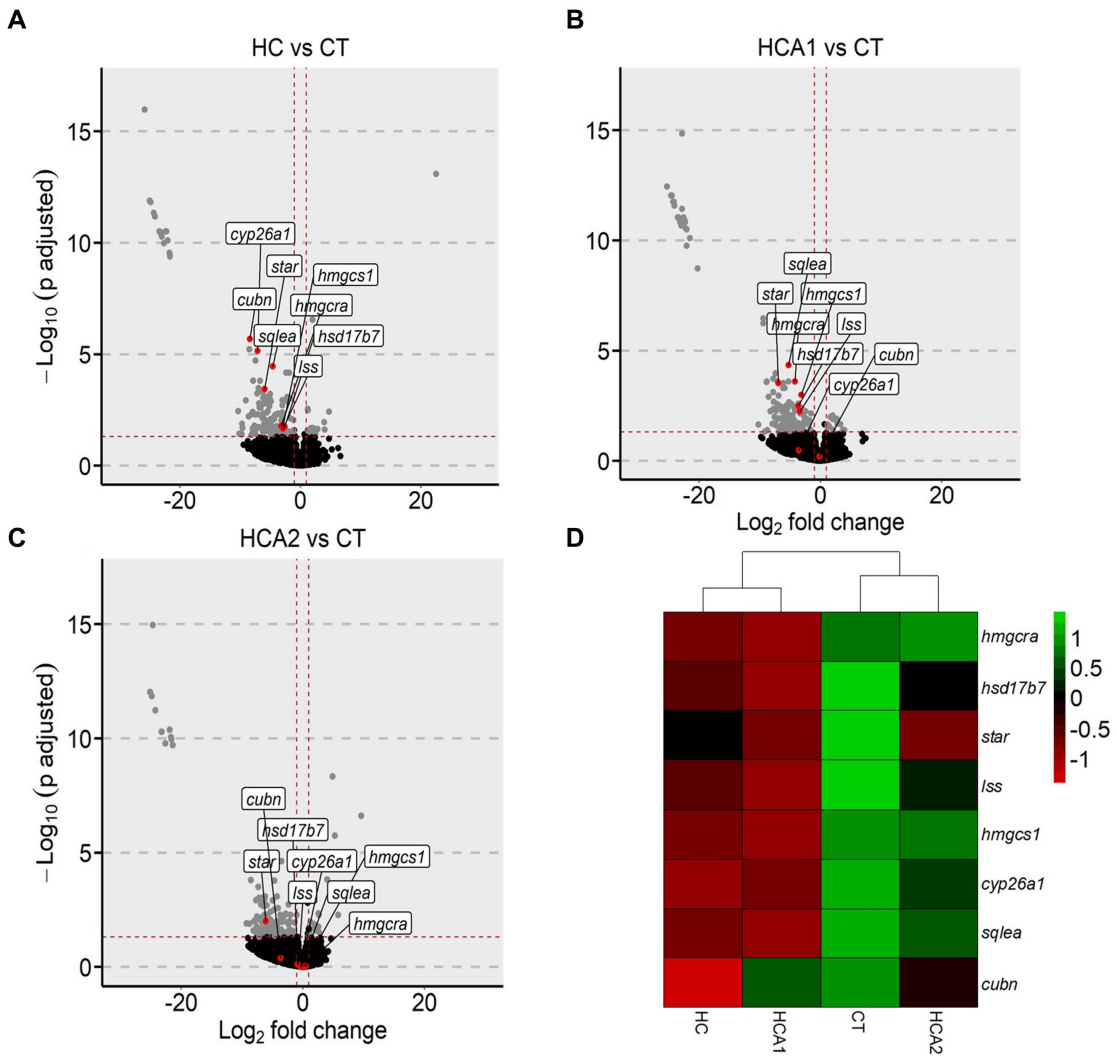
Alteration of genes related to cholesterol biosynthesis in zebrafish fed high cholesterol diet with and without microbial oil. Volcano plots highlighting the fold-changes in the intestinal cholesterol biosynthesis-related genes in **(A)** HC vs. CT, **(B)** HCA1 vs. CT and **(C)** HCA2 vs. CT transcriptome comparisons. Heatmap **(D)** showing hierarchical clustering of CT, HC, HCA1 and HCA2 groups. Each treatment group consisted of at least five biological replicates.

We first studied the influence of the HCA2 diet on the expression of all the genes that were altered by the high cholesterol diet, i.e., which were differentially expressed in HC vs. CT comparison. Of the 146 differentially downregulated genes in the HC vs. CT comparison, the normalized counts of 13 genes were increased in the HCA2 group (Supplementary Figure 5). Many of these 13 genes are involved in cholesterol biosynthesis. We selected 8 genes that were significantly downregulated in the HC vs. CT comparison (Figure 8A). In the HCA1 group, 6 out of the aforementioned 8 genes were significantly downregulated, compared to the CT group (Figure 8B). However, in the HCA2 group, only one gene was significantly downregulated and 7 were not differentially expressed (Figure 8C) compared to the CT group indicating a possible effect on cholesterol biosynthesis. Hierarchical clustering of the normalized counts of the genes revealed that the gene expression profile of HCA2 group was similar to the CT group (Figure 8D).

#### 3.3.3. HCA2 diet increased the expression of genes involved in lipoprotein metabolism

**Figure 9:**
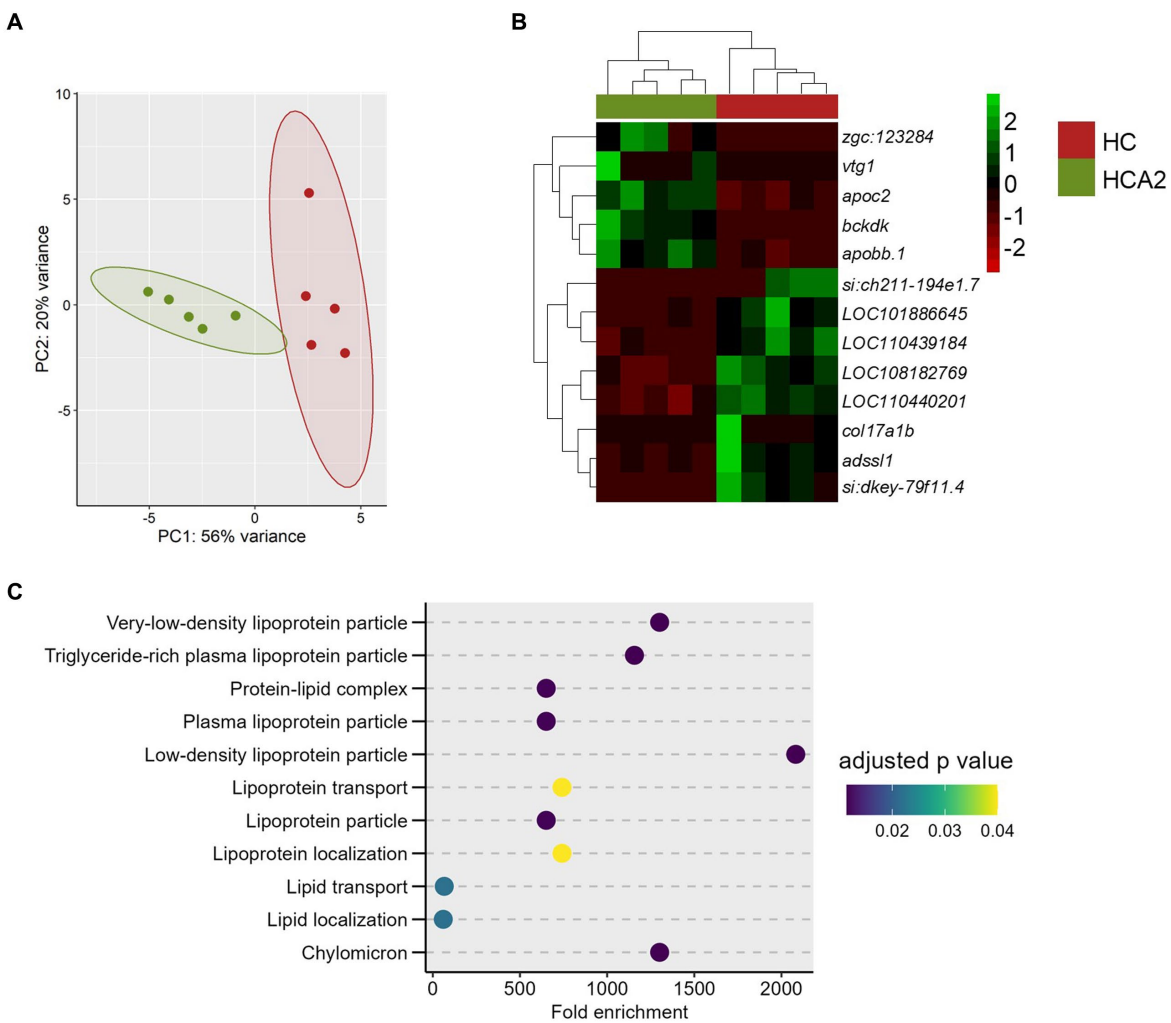
Differences in the transcriptome of the HCA2 and HC groups. Principal component analysis **(A)** and Hierarchical clustering **(B)** of the differentially expressed genes (DEGs) in the HCA2 group compared to the HC group. Transcripts with an adjusted *p* value (Benjamini-Hochberg method) < 0.05 and |Log_2_ fold change| ≥ 1 were considered significantly differentially expressed. Dot-plot **(C)** showing the enriched GO terms, by considering the upregulated genes in the HCA2 vs. HC group comparison. Each treatment group consisted of at least five biological replicates.

We also performed a comparison of the transcriptome of the HCA2 and HC groups (Figure 9A). Thirteen DEGs were identified, of which 5 were upregulated and 8 were downregulated in the HCA2 group (Figure 9B; Supplementary Table 10). The GO enrichment analysis of the upregulated DEGs revealed the enrichment of terms like chylomicron, lipoprotein transport and lipid transport (Figure 9C).

### 3.4. HCA2 diet altered the plasma lipidome of the zebrafish model

**Figure 10:**
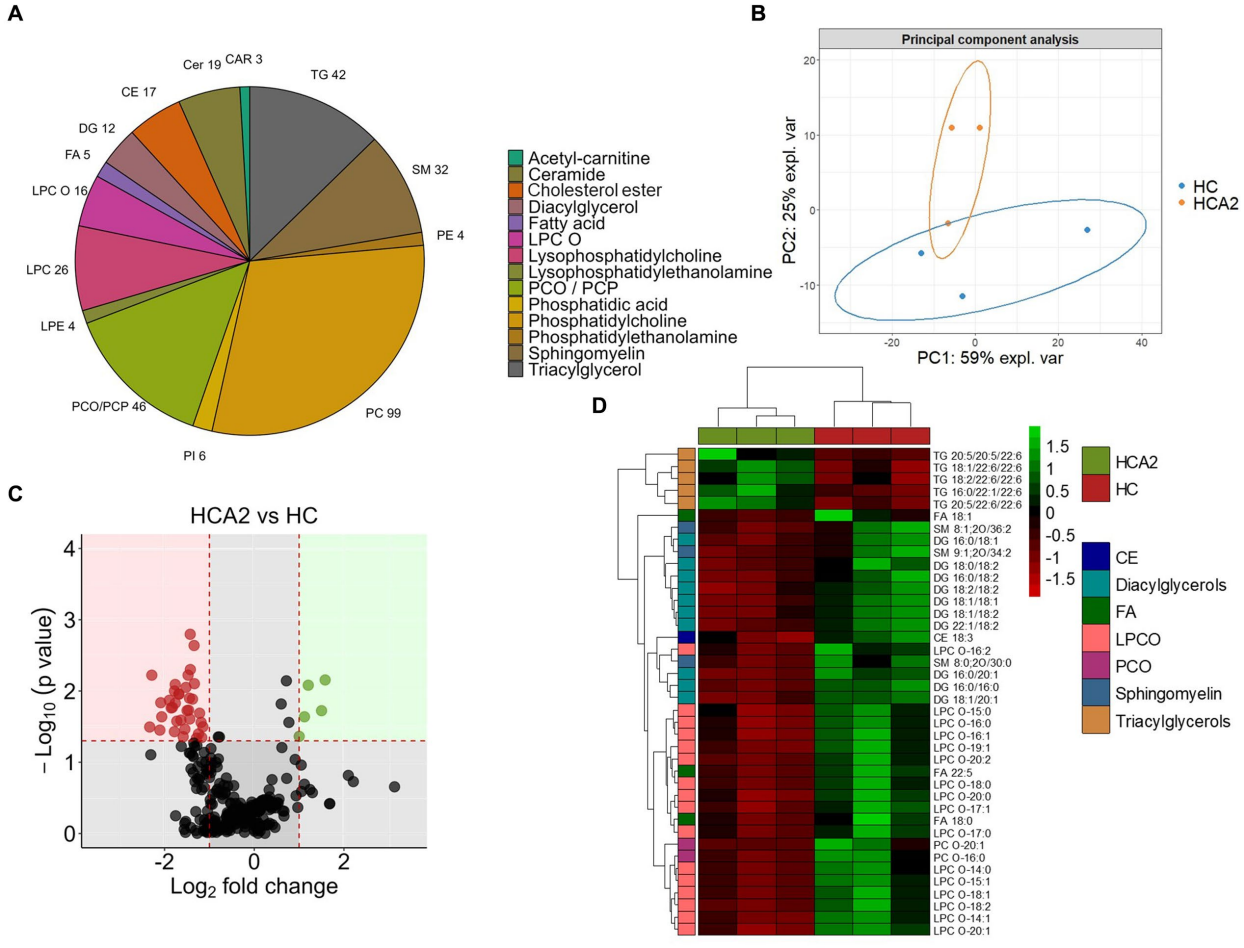
Differences in the lipid classes and species in the plasma of zebrafish fed the high cholesterol diet with and without microbial oil for a period of 12 weeks. Pie chart **(A)** of lipid classes in the plasma of zebrafish fed the high cholesterol (HC) diet and microbial oil supplemented (HCA2) diet. PCA **(B)** of the lipidome data from the 6 plasma samples that were used for the analysis (*n* = 3 per group). The first two components captured 59 and 25% of the variation of the data, respectively. **(C)** Volcano plot illustrating the differentially abundant (|Log_2_ fold-change| ≥ 1, *p* value < 0.05) lipid species in the HCA2 vs. HC diet group comparison. Red dots indicate downregulated and green dots indicate upregulated lipid species in the HCA2 group compared to the HC group. Heatmap **(D)** showing diet-group based hierarchical clustering of 40 differentially abundant lipid species.

**Figure 11:**
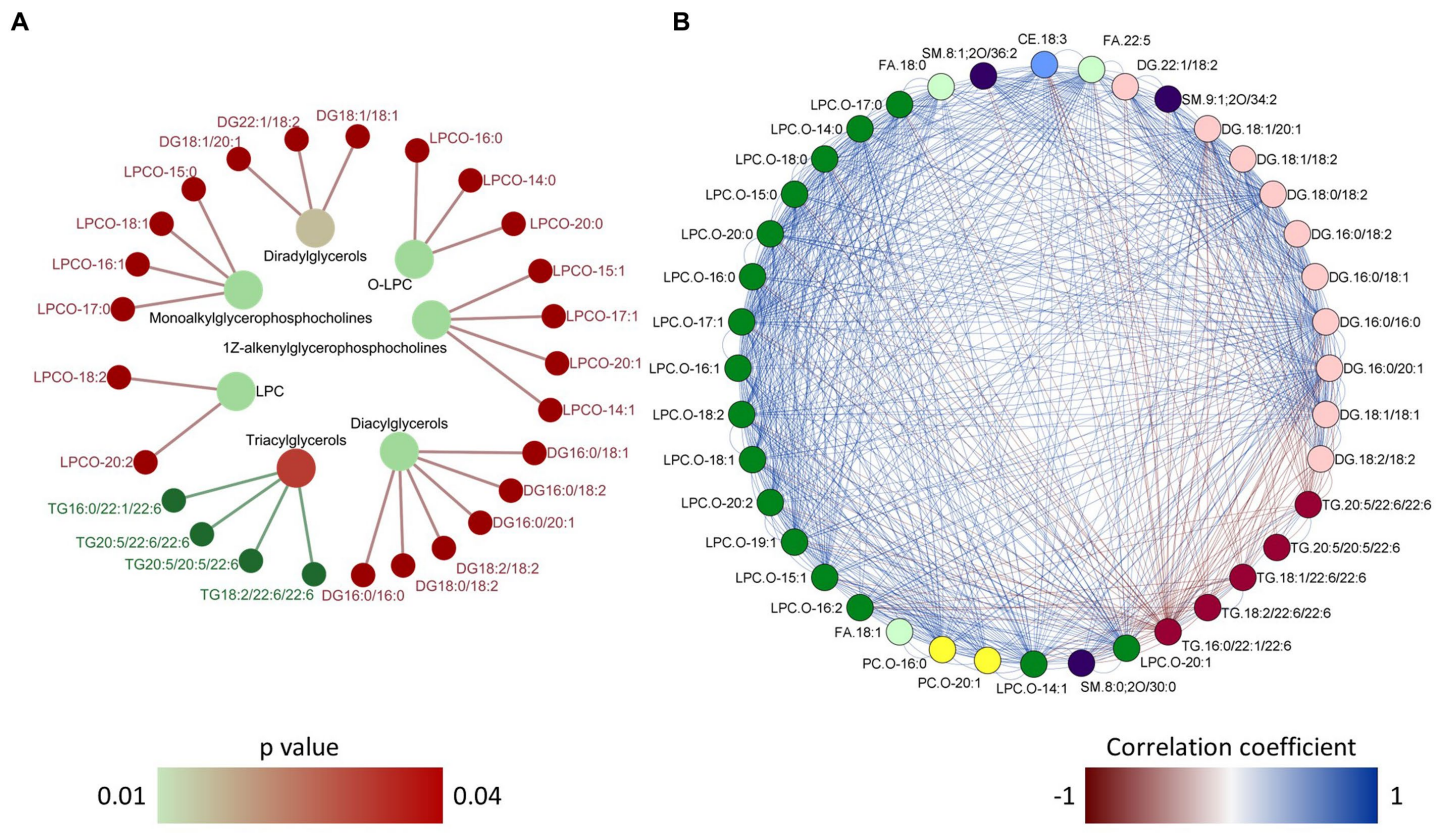
Over representation analysis and correlation network of the differentially abundant lipid species. Network plot **(A)** showing the link between the enriched lipid classes and differentially abundant lipid species in the plasma of zebrafish. Over representation analysis was performed using a cut-off of *p* value < 0.05 after adjusting for multiple testing. The color of the nodes in the outer circle indicate the lipid species that had higher and lower abundance in the HCA2 group compared to the HC group. The gradient color varies with the *p* value for each enriched lipid class. Network plot **(B)** showing the correlation between the 40 differentially abundant lipid species. The color of the edges indicate positive (blue) or negative correlation. A particular lipid class is color coded and only the links with correlation coefficient greater than 0.75 or less than 0.75 and *p* value of < 0.05 are shown in the network.

In order to gain deeper insights into the impact of dietary microbial oil supplementation on lipid species in plasma, we compared the global lipidomic profile of the HCA2 group with the HC group. The 331 lipid species annotated at level 1 belonged to 14 different lipid classes (Figure 10A; Supplementary Table 11) and they were used for further analysis. Unsupervised principal component analysis revealed a clear group-based clustering of the samples (Figure 10B). Subsequently, 40 plasma lipid species were identified as significantly altered in the HCA2 group compared to the HC group (Figure 10C; Supplementary Table 11). These 40 significantly altered lipids belonged to 7 classes. Among these were 5 species of TAGs that had significantly higher abundance. On the other hand, 35 species which included cholesterol ester (CE), diacylglycerols (DAG), free fatty acids (FA), alkyl lysophospholipids (LPCO), alkyl phosphatidylcholines (PCO) and sphingomyelines had significantly lower abundance in the HCA2 group compared to HC group (Figure 10D). The HCA2 group had significantly increased TAGs with 22:6 or 20:5 fatty acids. On the other hand, the diacylglycerols which had lower abundance were rich in 18:1, 16:0, 16:1 and 18:2 fatty acids. Furthermore, cholesterol ester 18:3 was also significantly reduced in the HCA2 group. ORA of the significantly altered lipids revealed an enrichment of the TAGs, lysophosphatidylcholines, diradylglycerols and diacylglycerols in the plasma of the HCA2 group (Figure 11A). A correlation network analysis revealed a high correlation between several differentially abundant lipid species. We found that the differentially abundant diacylglycerols, lysophospholipids and phosphatidylcholines were positively correlated. The TAGs were positively correlated among themselves. On the other hand, most of the other differentially abundant lipid species and the differentially abundant TAGs were negatively correlated (Figure 11B).

## 4. Discussion

An unhealthy diet is a critical risk factor that contributes to the development of CVDs (69). While a Western diet can increase CVD-related fatality, Mediterranean diets rich in omega-3 fatty acids effectively mitigate hyperlipidemia and CVDs (70). With their differential action on transcription factors, the omega-3 fatty acids, EPA and DHA may have independent positive effects on the cardiovascular system (71). Most studies on human and animal models investigated the effects of omega-3 fatty acids in fish oil on TAGs and lipoproteins (72, 73). *Schizochytrium* sp. are marine heterotrophic microorganisms rich in n-3 PUFAs. *Schizochytrium* oil contains about 15 and 39% of EPA and DHA, respectively compared to 14.9 and 13% in fish oil and the EPA:DHA ratio in *Schizochytrium* oil is 0.4 and that in fish oil is 1.1 (42, 43). There are several studies which have evaluated the effect of *Schizochytrium* against dyslipidemia. A systematic analysis revealed that marine microorganism-derived oils can reduce the circulating TAG and increase HDL cholesterol in humans (74). But these human studies have been limited to blood lipid values and have not explored the deeper mechanisms of action in the intestine and liver. *Schizochytrium* oil and fish oil could reduce the levels of total cholesterol and LDL cholesterol in the plasma of rats, but the triglyceride content was found to reduce only in rats that consumed *Schizochytrium* oil. This microbial oil also increased the expression of genes such as *Insulin-induced gene 1* (*Insig-1*) and *LDL recepto*r (*Ldlr*) in the liver of rats, whereas the fish oil could only induce the expression of the latter gene (40). However, *Schizochytrium*-derived oil is also rich in pro-atherogenic palmitic acid which is about 30% of the total fatty acids (42). To understand the effectiveness of the whole oil in preventing CVDs, we used the zebrafish model of hypercholesterolemia, which is characterized by key biomarkers like elevated plasma lipid species and aberrated lipid metabolism (64). We focused on the effect of dietary microbial oil supplementation on the liver, mid-intestine and plasma parameters. The results indicate that the tested high level of the microbial oil can keep the levels of plasma cholesterol and lipoproteins in check. Intestinal transcriptome comparisons also pointed to the effectiveness of microbial oil in alleviating the effects of hypercholesterolemia. Plasma lipidomic analysis revealed a significant increase in the LC-PUFAs of the TAG. This study provides insights into the mechanisms of mitigation of hypercholesterolemia by *Schizochytrium* oil.

### 4.1. Microbial oil affected the plasma lipid species and hepatic gene expression and vacuolization

We found that a higher level of microbial oil can lower the plasma TC, LDL-C and TAG levels in zebrafish fed a high-cholesterol diet. Plasma TC correlated positively with LDL-C but not with HDL-C levels. Hence, the reduction in TC was likely driven by the decreased plasma LDL-C of the HCA2 group, probably due to the uptake of LDL-C by the peripheral tissues (75). Studies in humans have indicated that elevating the HDL-C concentration without decreasing triglycerides may not prevent CVDs (76). We observed both higher plasma HDL and lower TAG content in the HCA2 group compared to the HC group. There are several mechanisms by which DHA can restore dyslipidemia, including stimulation of β-oxidation (58) and reverse cholesterol transport. We found that the expression of *cpt1aa*, which encodes a mitochondrial transmembrane enzyme required for beta oxidation, was higher in the liver of the HCA2 group. We also found significantly higher amounts of HDL cholesterol in the plasma and increased mRNA levels of *lcat* and *scarb1* in the liver of the HCA2 group. The activity of LCAT and SCARB1 is critical for reverse cholesterol transport, a mechanism by which the body removes excess cholesterol by delivering it to liver (26). Therefore, the increase in plasma HDL and increased expression of *cpt1aa*, *lcat* and *scarb1* in the liver of the HCA2 group may have contributed to reduced circulating TAG. Diets rich in lipids can increase the circulating HDL cholesterol by increasing its biosynthesis and reducing the breakdown rate, as reported in a rat study (77) and human studies (78, 79). However, we did not find any significant increase in HDL concentration in the plasma of zebrafish after feeding a high cholesterol diet. Our previous study also confirmed that plasma HDL concentration in zebrafish remains unaltered after a high cholesterol feeding (64). Like other teleosts, the plasma of zebrafish has an HDL dominant lipoprotein profile (80). Nevertheless we find reports of increase in HDL fraction after feeding with a high cholesterol diet (51).

Although abnormal levels of circulating lipoproteins are considered indicators of CVD development, these parameters are not ideal CVD biomarkers. Ratios of plasma lipoproteins are regarded as better suited in predicting cardiovascular risk in humans (81). In our study, the plasma Castelli risk indices (I and II), atherogenic coefficient and atherogenic indices were significantly improved in the HCA2 group. These ratios are considered reliable parameters in predicting cardiovascular diseases (82, 83). We did not find any significant correlation between TAG:HDL-C ratio and LDL-C content of plasma. However, the Castelli I index was significantly correlated with the LDL-C values. When consumed excessively, lipids will accumulate in the liver (84). Excess cholesterol is accumulated as lipid droplets in hepatocytes, through the action of enzymes located mainly in their endoplasmic reticulum (85), as observed in humans (86) as well as several model species (87, 88). Such excess accumulation of lipids in the liver can lead to lipotoxicity (89). The associated abnormal responses include organellar dysfunction (90), abnormal activation of intracellular signaling pathways (91), chronic inflammation (92) and apoptosis (17, 93). In the present study, 6 month-old zebrafish had significantly more hepatic vacuoles when fed high cholesterol than the control diet. This outcome was not evident in our previous study with 1-year-old zebrafish (64), probably because liver vacuolization increases with age (94, 95). Hence, 6-month-old adult zebrafish can be considered a suitable model for understanding the effects of hypercholesterolemia. The application of two levels of dietary microbial oil resulted in vacuolization to the same extent as noted in the control group. A previous study has indicated that ≥ 0.5% DHA can increase β-oxidation in the liver of zebrafish (96) which will lead to reduced vacuolization. Increase in the expression of the SR-BI is also associated with a reduction in liver vacuolization (97, 98). In our study, β-oxidation-associated gene *cpt1aa*, and the HDL metabolism-linked gene *scarb1* were upregulated in the HCA2 diet group but not in the HCA1 group, even though vacuolization was reduced in both the groups compared to the HC group. This indicates an alternate vacuole-reduction mechanism in the HCA1 group compared to the abovementioned alteration in the HCA2 group.

### 4.2. High level of microbial oil can favorably maintain cholesterol metabolism and strengthen antioxidant capacity

In the present study, the HCA1 diet had 3.1% and the HCA2 diet had 6.6% of the microbial oil. We did not study the effect of inclusion of more than 6.6 g of *Schizochytrium* oil/100 g feed. Toxicological studies have not revealed any adverse effects of *Schizochytrium* in rats and pigs, when fed at 3,343 mg/kg/day and 1,121 mg/kg/day, respectively (99, 100). In the present study, *Schizochytrium* oil was fed at a rate of 2,640 mg/kg/day to zebrafish. Furthermore, the oil is considered safe for human consumption (101). Although both diets prevented lipid infiltration into the liver, only the blood lipid profile of the HCA2 group was similar to that in the CT group. As for the genes linked to cholesterol biosynthesis, the intestinal expression of 2 and 7 genes in the HCA1 and HCA2 groups, respectively, was similar to those in the CT group. Compared to the CT group, the HC group had higher plasma TC and LDL-C but caused a suppression of genes linked to cholesterol biosynthesis in the intestine. On the other hand, the lower plasma TC and LDL-C levels along with the unaltered expression of cholesterol biosynthesis genes in the HCA2 group is likely pointing to a greater efficacy of the higher level of dietary microbial oil. This proposition is strengthened by the observation on the upregulation of genes linked to lipoprotein transport, lipid transport and chylomicron in the HCA2 group compared to the HC group, notably due to the upregulation of *apobb.1* and *apoc2* genes in the HCA2 group. The protein coded by the *apobb.1* gene in the intestine is the carrier of absorbed neutral lipids, cholesteryl esters, and TAGs. Templehof et al. (102) revealed that the deletion of *apob* genes in zebrafish can increase lipid infiltration into the liver. The protein coded by *apoc2* is an activator of the lipoprotein lipase enzyme which is in turn needed for the hydrolysis of plasma TAGs, thereby clearing TAGs in circulation. In zebrafish, loss of *apoc2* can lead to hyperlipidemia (103). The upregulation of the expression of *apobb.1* and *apoc2* genes along with the unaltered expression of cholesterol biosynthesis genes in the HCA2 group indicate the need for a high level of microbial oil to counter hyperlipidemia. Microbial oil (HCA2 diet group) caused alteration of genes revealed the enrichment of KEGG pathways like glutathione metabolism, drug metabolism-cytochrome p450 and metabolism of xenobiotic by cytochrome p450. These pathways were enriched because of the upregulation of *microsomal glutathione S-transferase 1.1* and *microsomal glutathione S-transferase 1.2* genes in the intestine of zebrafish. Overexpression of microsomal glutathione S-transferase can provide protection against cytotoxicity and oxidative stress (104). Fish oil supplementation, which is also a rich source of long chain PUFAs, can increase the gene expression levels of glutathione transferases to defend against reactive oxygen species production (105). Therefore, our results suggest that the inclusion of microbial oil in the diet activates the antioxidant system in the intestine possibly to combat inflammation and oxidative stress caused by the high cholesterol diet (52, 106).

### 4.3. Changes in lipidomic profiles mark the protective effect of microbial oil

Lipid-rich diets can cause dyslipidemia that leads to the development of CVDs (107, 108). Recent lipidomic studies on zebrafish have identified many tissue specific lipids; 508 lipids in the liver cells of zebrafish (109), 898 lipids in 7 days old larvae (110) and 2,112 lipids in the right optic nerve of adult zebrafish (111). To our knowledge, this is the first study on the plasma lipidome of zebrafish, reporting 331 lipid species. Most of the 40 differently altered lipid species had lower abundance in the HCA2 group and 5 lipid species of the class TAGs had higher abundance in the HCA2 group compared to the HC group. While reduced serum n-3 PUFA is a CVD risk factor (112), higher circulating DHA levels can lower the risk (113). Dietary omega-3 PUFAs were found to increase n-3 PUFA incorporated plasma and liver phosphatidylcholine, lysophosphatidylcholine, and cholesteryl esters (37). Other studies have identified triglycerides with fewer double bonds and TAGs rich in stearic acid, a saturated fatty acid, as strong predictors of cardiovascular events (114, 115). In our study, the 5 species of triacylglycerols that had higher abundance in the plasma of the HCA2 group were rich in C22:6 and C20:5 fatty acids. Similarly, the proportion of plasma triglycerides containing LC-PUFAs was increased in humans who consumed fish oil (38). Our results indicate a possible dietary microbial oil-induced increase in the LC-PUFA-rich plasma TAGs. Docosapentaenoic acid (C22:5), stearic acid (C18:0) and oleic acid (C18:1) were the three free fatty acids that had significantly lower abundance in the HCA2 group compared to the HC group. Although free stearic acid and oleic acid are generally not considered pro-atherogenic fatty acids (116), EPA + DHA consumption can reduce the content of these fatty acids in plasma of humans (117). We also found a significant reduction in the different species of alkyl lysophosphatidylcholines (LPCO) in the HCA2 group. These molecules are involved in a broad range of physiological processes and increased LPC levels are biomarkers of dysregulated lipid metabolism (118, 119). Hence, the reduced LPCO levels compared to the corresponding values in the HC group reflects a normal lipid metabolism in the HCA2 group. Several diacylglycerols (DAGs) containing C16 and C18 fatty acids also had lower abundance in the plasma of the HCA2 group. Although, early reports have indicated that dietary DAGs can be beneficial to prevent dyslipidemia (120, 121), circulating levels of DAGs are linked to specific diseases. For instance, liver diseases are associated with increase in plasma C18:1 and C16:1 containing DAGs (122). Furthermore, in humans, metabolic syndrome was correlated with higher plasma C14:0, C16:0 and C18:0 containing DAGs (123). As observed in our study, fish oil supplementation was found to alter the DAG levels in high fat diet fed rats (124). Some lipid species like sphingomyelin, SM 8:1;2O/34:2 and LPC C18:1 have been reported as risk factors of cardiovascular diseases (114). The reduction of these key lipid species in the plasma of zebrafish indicates the effectiveness of microbial oil supplementation against dyslipidemia.

### 4.4. Microbial oil may not prevent the alteration of cytoskeleton organization

Transcriptomic analyses revealed a consistent enrichment of GO terms linked to microtubule organization based on the downregulated DEGs in the HC, HCA1 and HCA2 groups compared to the CT group. Microtubules, the polarized filament proteins which form the cytoskeleton, are critical for maintaining the polarity of enterocytes (125). Cholesterol is an integral part of the plasma membrane, and it also contributes to the apical polarity of the enterocytes (126). In addition, cholesterol is part of membrane microdomains termed lipid rafts that also contain saturated phospholipids and sphingolipids including glycolipids and sphingomyelin. Cholesterol-enriched rafts are required for cytoskeleton rearrangements (127). Several lines of evidence have indicated that changes in cholesterol metabolism may affect membrane-cytoskeleton interactions (128, 129). In line with our finding, other studies have also documented cholesterol-induced alteration of the abundance of cytoskeletal proteins (130, 131). It seems that both levels of microbial oil were unable to abate the dietary cholesterol-induced suppression of cytoskeletal genes. This indicates that dietary cholesterol imparts a negative effect on the cytoskeletal elements despite the intervention with the microbial oil. Therefore, a deeper understanding of the impact of cholesterol on the cytoskeletal organization is needed to unravel the tenacious effects of hypercholesterolemia.

## 5. Conclusion

Taking advantage of a zebrafish hypercholesterolemic model, we demonstrated how a novel EPA and DHA-rich microbial oil could control the negative effects of a high-cholesterol diet. *Schizochytrium*-derived oil impacted the expression of genes involved in lipid metabolism in the liver. Plasma lipidomic profiling revealed the efficacy of the microbial oil in increasing the LC-PUFA content of triacylglycerol species, lowering of the alkyl lysophosphatidylcholine species and several diacylglycerols. The dietary microbial oil-based approach demonstrated through this study, mainly to tackle disrupted cholesterol metabolism, holds promise for a vast majority of the human population afflicted by CVD-associated risks.

## Data availability statement

The datasets presented in this study can be found in online repositories. The names of the repository/repositories and accession number(s) can be found below: https://www.ncbi.nlm.nih.gov/, Sequence Read Archive, PRJNA944406.

## Ethics statement

The approval for the conduct of this study was obtained from the Norwegian Animal Research Authority (FDU ID: 22992).

## Author contributions

VK, JD, and AG: study design. JD: feed preparation. SR and AG: feeding experiment. SR: qPCR analysis. AG: histological analysis. SR and AG: bioinformatic data analysis. AG and VK wrote the manuscript. JF, PO, and MS edited the manuscript. All authors contributed to the article and approved the submitted version.

## Funding

SR and AG were supported by Netaji Subhas-ICAR International Fellowships (NS-ICAR IFs) from the Indian Council of Agricultural Research, India.

## Conflict of interest

JD was employed by SPAROS Lda.

The remaining authors declare that the research was conducted in the absence of any commercial or financial relationships that could be construed as a potential conflict of interest.

The handling editor SM declared a past collaboration with the author VK.

## Publisher’s note

All claims expressed in this article are solely those of the authors and do not necessarily represent those of their affiliated organizations, or those of the publisher, the editors and the reviewers. Any product that may be evaluated in this article, or claim that may be made by its manufacturer, is not guaranteed or endorsed by the publisher.
