# Supplementary material for: Protective mechanisms of a microbial oil against hypercholesterolemia: evidence from a zebrafish model
Source: Front Nutr. 2023 Jun 26;10:1161119. doi: 10.3389/fnut.2023.1161119 (PMC10332275; doi:10.3389/fnut.2023.1161119)
Supplement: Supplementary file 2 [file Presentation_1.pptx]

## Slide 1
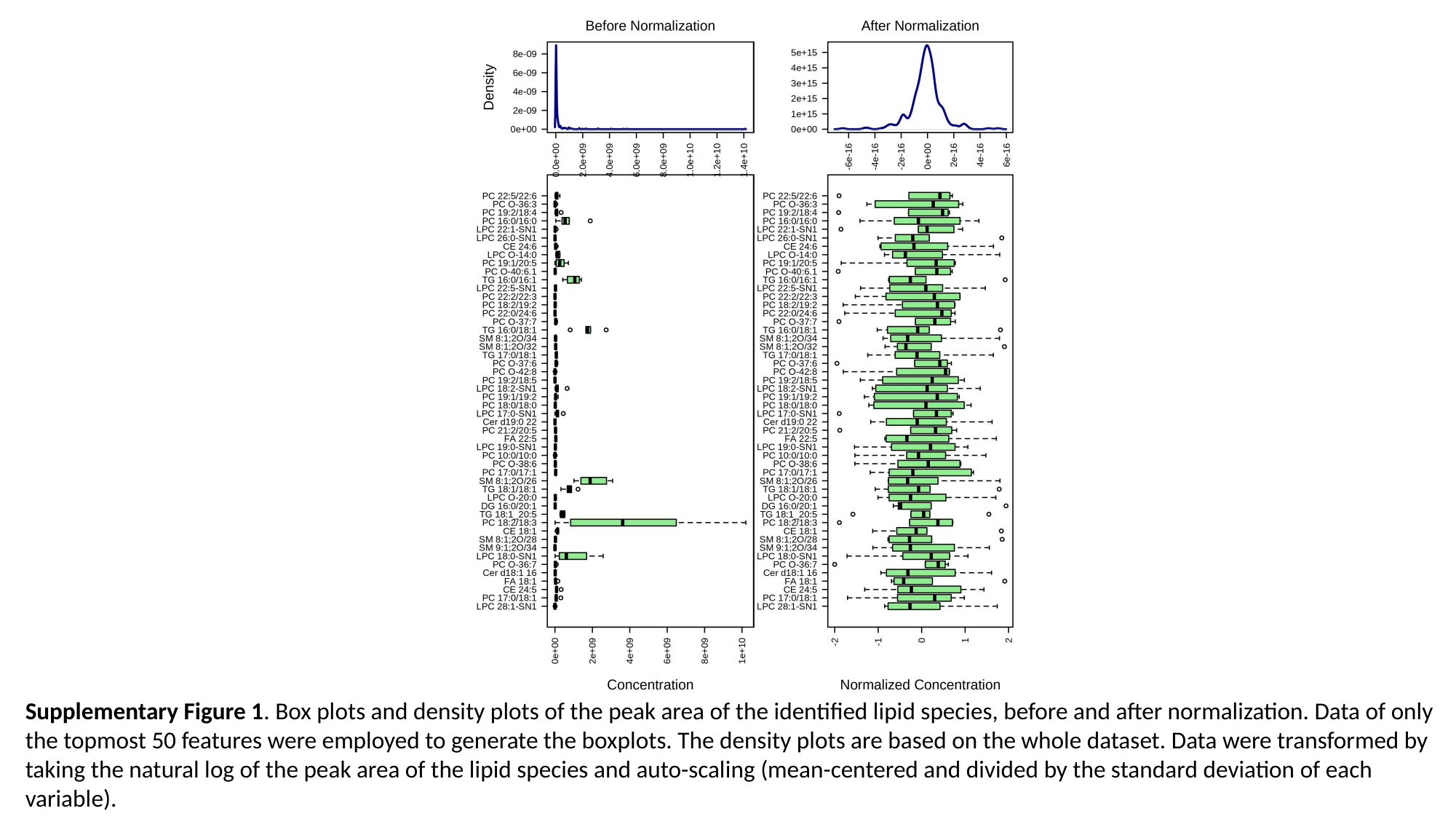

Supplementary Figure 1. Box plots and density plots of the peak area of the identified lipid species, before and after normalization. Data of only the topmost 50 features were employed to generate the boxplots. The density plots are based on the whole dataset. Data were transformed by taking the natural log of the peak area of the lipid species and auto-scaling (mean-centered and divided by the standard deviation of each variable).

## Slide 2
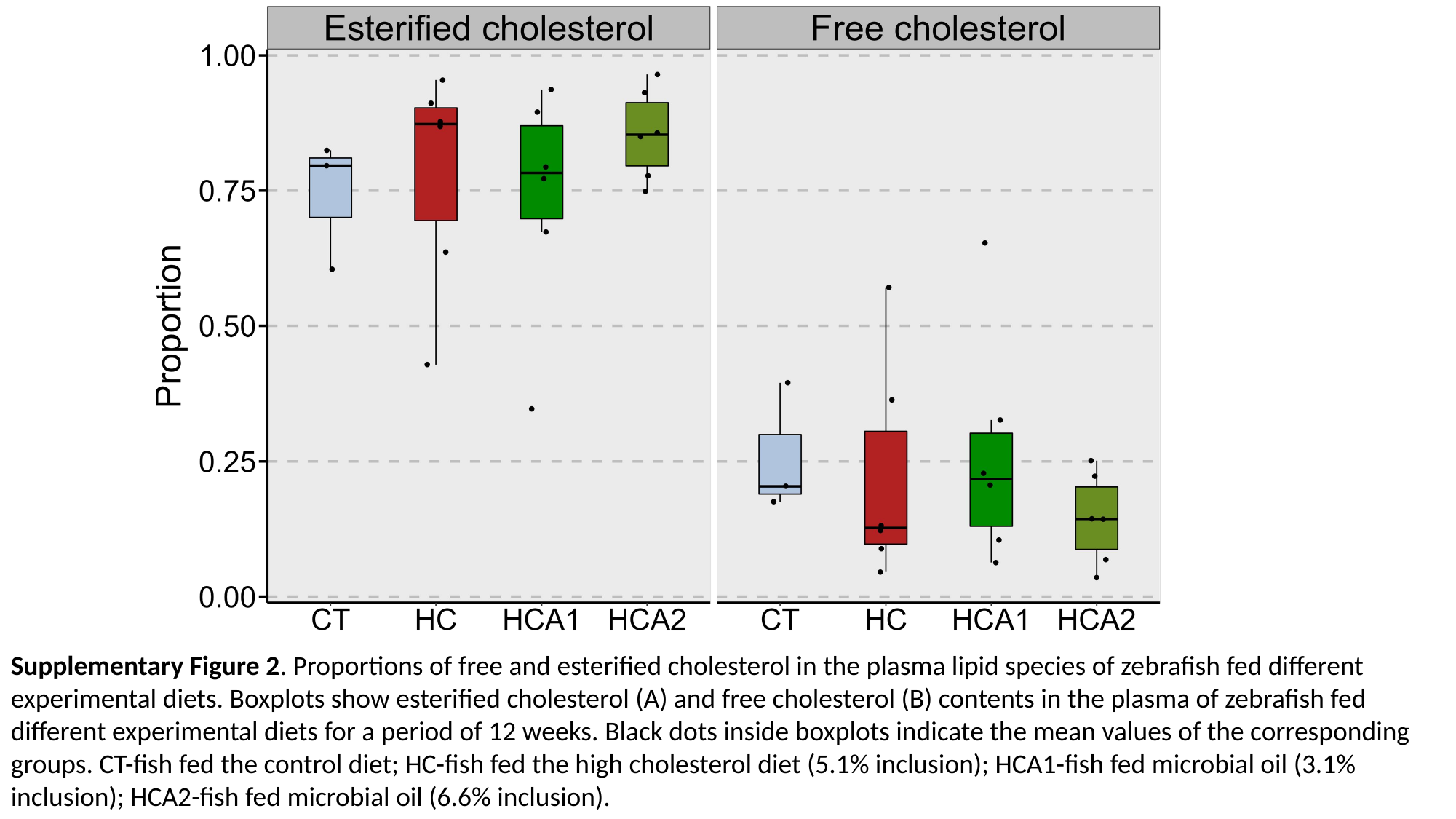

Supplementary Figure 2. Proportions of free and esterified cholesterol in the plasma lipid species of zebrafish fed different experimental diets. Boxplots show esterified cholesterol (A) and free cholesterol (B) contents in the plasma of zebrafish fed different experimental diets for a period of 12 weeks. Black dots inside boxplots indicate the mean values of the corresponding groups. CT-fish fed the control diet; HC-fish fed the high cholesterol diet (5.1% inclusion); HCA1-fish fed microbial oil (3.1% inclusion); HCA2-fish fed microbial oil (6.6% inclusion).

## Slide 3
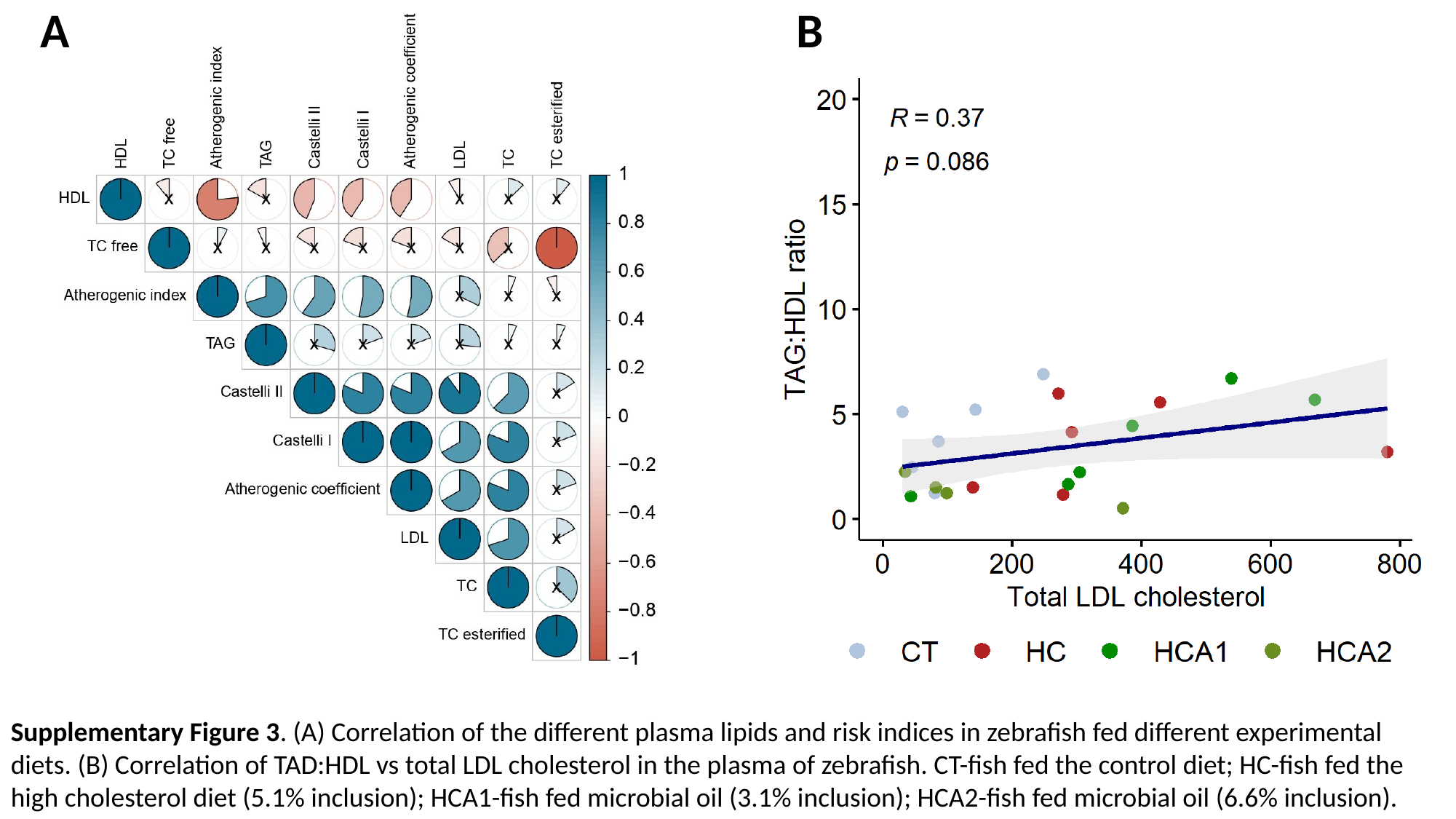

B
A
Supplementary Figure 3. (A) Correlation of the different plasma lipids and risk indices in zebrafish fed different experimental diets. (B) Correlation of TAD:HDL vs total LDL cholesterol in the plasma of zebrafish. CT-fish fed the control diet; HC-fish fed the high cholesterol diet (5.1% inclusion); HCA1-fish fed microbial oil (3.1% inclusion); HCA2-fish fed microbial oil (6.6% inclusion).

## Slide 4
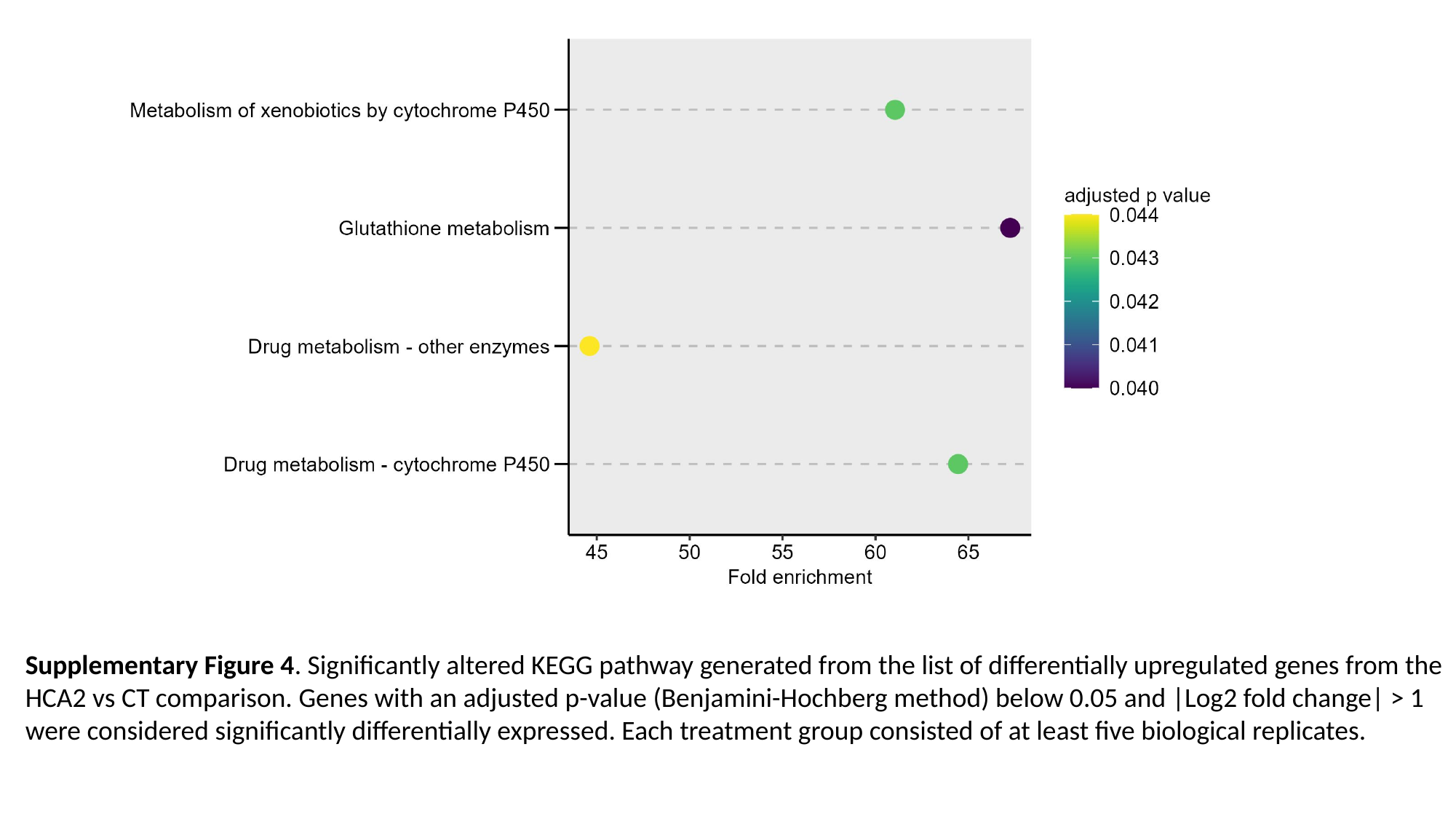

Supplementary Figure 4. Significantly altered KEGG pathway generated from the list of differentially upregulated genes from the HCA2 vs CT comparison. Genes with an adjusted p-value (Benjamini-Hochberg method) below 0.05 and |Log2 fold change| > 1 were considered significantly differentially expressed. Each treatment group consisted of at least five biological replicates.

## Slide 5
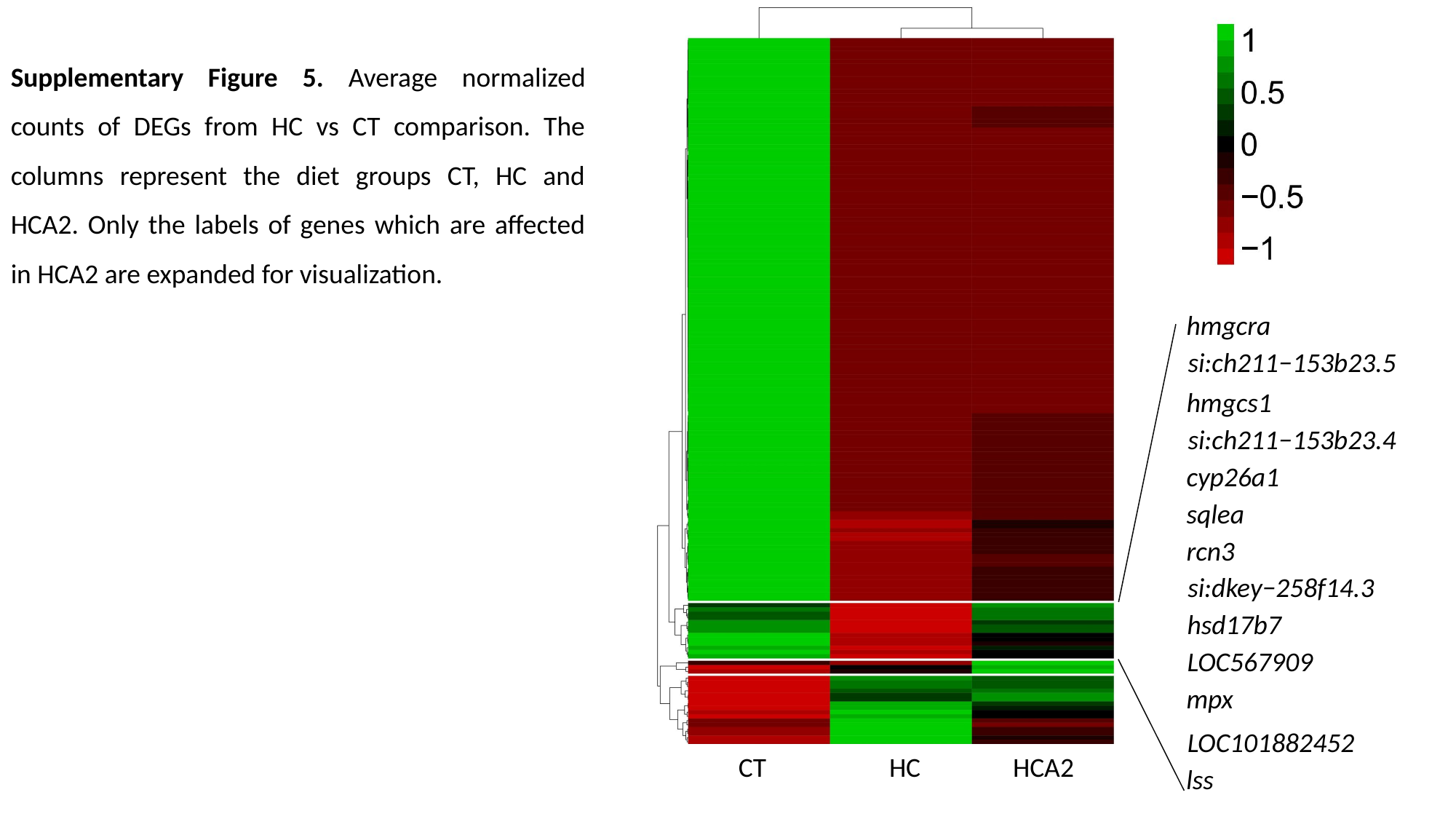

hmgcra
si:ch211−153b23.5
hmgcs1
si:ch211−153b23.4
cyp26a1
sqlea
rcn3
si:dkey−258f14.3
hsd17b7
LOC567909
mpx
LOC101882452
CT
HC
HCA2
lss
Supplementary Figure 5. Average normalized counts of DEGs from HC vs CT comparison. The columns represent the diet groups CT, HC and HCA2. Only the labels of genes which are affected in HCA2 are expanded for visualization.
